# A metabolic signature of long life in *Caenorhabditis elegans*

**DOI:** 10.1186/1741-7007-8-14

**Published:** 2010-02-10

**Authors:** Silke Fuchs, Jacob G Bundy, Sarah K Davies, Jonathan M Viney, Jonathan S Swire, Armand M Leroi

**Affiliations:** 1 Division of Biology, Silwood Park Campus, Imperial College London, SL5 7PY, UK; 2 Current address: Division of Cell and Molecular Biology, South Kensington Campus, Imperial College London, London SW7 2AZ, UK; 3 Biomolecular Medicine, Department of Surgery and Cancer, Faculty of Medicine, South Kensington Campus, Imperial College London, London SW7 2AZ, UK; 4 National Heart and Lung Institute, South Kensington Campus, Imperial College, London, London SW7 2AZ, UK; 5 Centre for Bioinformatics, Division of Molecular Biosciences, South Kensington Campus, Imperial College London, London SW7 2AZ, UK

## Abstract

**Background:**

Many *Caenorhabditis elegans *mutations increase longevity and much evidence suggests that they do so at least partly via changes in metabolism. However, up until now there has been no systematic investigation of how the metabolic networks of long-lived mutants differ from those of normal worms. Metabolomic technologies, that permit the analysis of many untargeted metabolites in parallel, now make this possible. Here we use one of these, ^1^H nuclear magnetic resonance spectroscopy, to investigate what makes long-lived worms metabolically distinctive.

**Results:**

We examined three classes of long-lived worms: dauer larvae, adult Insulin/IGF-1 signalling (IIS)-defective mutants, and a translation-defective mutant. Surprisingly, these ostensibly different long-lived worms share a common metabolic signature, dominated by shifts in carbohydrate and amino acid metabolism. In addition the dauer larvae, uniquely, had elevated levels of modified amino acids (hydroxyproline and phosphoserine). We interrogated existing gene expression data in order to integrate functional (metabolite-level) changes with transcriptional changes at a pathway level.

**Conclusions:**

The observed metabolic responses could be explained to a large degree by upregulation of gluconeogenesis and the glyoxylate shunt as well as changes in amino acid catabolism. These responses point to new possible mechanisms of longevity assurance in worms. The metabolic changes observed in dauer larvae can be explained by the existence of high levels of autophagy leading to recycling of cellular components.

See associated minireview: http://jbiol.com/content/9/1/7

## Background

The nematode *Caenorhabditis elegans *normally has a life-span of about three weeks. The dauer larva, however, lives for up to eight times longer [1]. In addition, mutations in scores of genes have been identified that increase longevity. These genes have been grouped into several pathways including the Insulin/Insulin-Like signalling pathway (IIS) [2-6], the dietary restriction pathway [7,8] and the translation control pathway [9], but how they regulate ageing individually and together is still obscure. What is certain, however, is that each of them influences the metabolism of the worm in some fashion. This has been shown by the discovery that particular longevity pathways control, or at least interact with, key regulators of metabolism [10-13] as well as many metabolic enzymes [14-22].

Despite these advances, our understanding of how altered metabolism influences longevity in worms, indeed, if it does so at all, remains very incomplete. In part, this is because attention has focused almost exclusively on the genes that control metabolism rather than metabolites themselves. Yet gene activity can only give a very dim outline of the activity of a metabolic network since much regulation occurs at the post-transcriptional, or even post-translational level, for example, by allosteric interactions among metabolites and the enzymes that catalyse them [23,24]. One way to investigate the activity of metabolic networks in a more direct fashion is metabolite profiling (also sometimes called metabolomics or metabonomics). Metabolomics has been previously combined with functional genomics to study a variety of biological problems and species [25-28] including, recently, *C. elegans *[29-31]. Here, we apply it to investigating the metabolic networks of a series of worms that are, for one reason or another, long-lived. Most of our long-lived worms are defective for components of the IIS pathway and one is translation defective; but we also study the dauer stage that forms when larvae are grown under stressful conditions. We show that all these long-lived worms have metabolic profiles that are not only very different from normal worms but also very similar to each other; in other words, that there is a *metabolic signature *for long-life in worms. The existence of this signature is surprising since the IIS and translation pathways are, at least, thought to influence longevity by quite distinct mechanisms [9,12]. This signature is composed of metabolites that function in several distinct parts of the network, including carbohydrate, amino acid and choline metabolism. Since our ultimate goal is an integrated model of worm metabolism, we also interrogate existing global gene expression data from *daf-2 *mutant worms [20] to give a general account of how the metabolic networks of long-lived worms differ from those with normal life-spans.

## Results and discussion

### Long-lived mutants have distinctive metabolic profiles

Of the various pathways known to regulate longevity in worms, the best known is the Insulin/Insulin-Like signalling (IIS) pathway [32,33]. Many mutations that disrupt components of this pathway affect the ability of larval worms to enter and leave the dauer stage, but they also increase the longevity and stress resistance of adults as well as reduce their fecundity [2-6,34].

**Figure 1:**
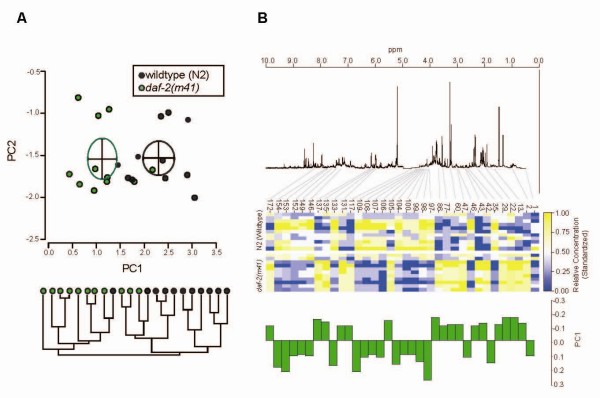
**The insulin-signalling mutant *daf-2(m41) *has a distinctive metabolic profile**. In this experiment *m41 *and N2 wild-type worms were initially raised at 15°C, transferred to 22.5°C at L4, and assayed at 240 hours post-bleaching. **A**. PCA (above) on the binned spectra shows that *daf-2(m41) *are clearly separable from N2 along PC1 (Means and 95% CI are given by ellipses); cluster analysis (below) separates the samples into two groups, one predominantly wild-type (8/9), the other predominantly *m41 *(10/13). The first three PCs respectively account for 50, 11 and 7% of the variance. **B**. Heatmap (middle) showing the standardized relative concentration of 34 bins out of 179 with a substantial (>0.1 and <-0.1) loading on PC1, their loads (below) and position on the ^+1^H NMR spectrum (above). The spectrum is the median of five N2 samples; intensities >5.0 ppm are scaled by a factor of 10.

We began by studying *m41*, a hypomorph mutation that disrupts *daf-2 *which encodes a tyrosine kinase that is expressed throughout the worm and is thought to act as a receptor for many of the 37 insulin-like ligands present in the *C. elegans *genome [35,36]. *daf-2(m41) *hermaphrodites are 10 to 90% longer-lived than wild-type worms [3,17,37,38] (our data not shown). Since *m41 *is a dauer-constitutive temperature-sensitive mutation we grew these worms at the permissive temperature, 15°C, until L4, transferred them to 22.5°C, and assayed their metabolites as old adults (240 hours). We did this by freezing the worms instantly in liquid nitrogen, extracting polar metabolites, and then acquiring ^1^H NMR spectra. The spectra showed a range of resonances from small molecule metabolites, typical of tissue extracts. We then divided the spectra into bins each chosen to represent as far as possible a single metabolite resonance. Principal components analysis (PCA) and hierarchical cluster analysis (HCA) of the reduced data showed that *daf-2(m41) *and wild-type samples have distinct metabolic profiles with little overlap between the two groups of samples in PC1 (Figure 1A). The loadings along this axis showed that many NMR-detectable metabolites contribute to the difference between the genotypes (Figure 1B).

**Figure 2:**
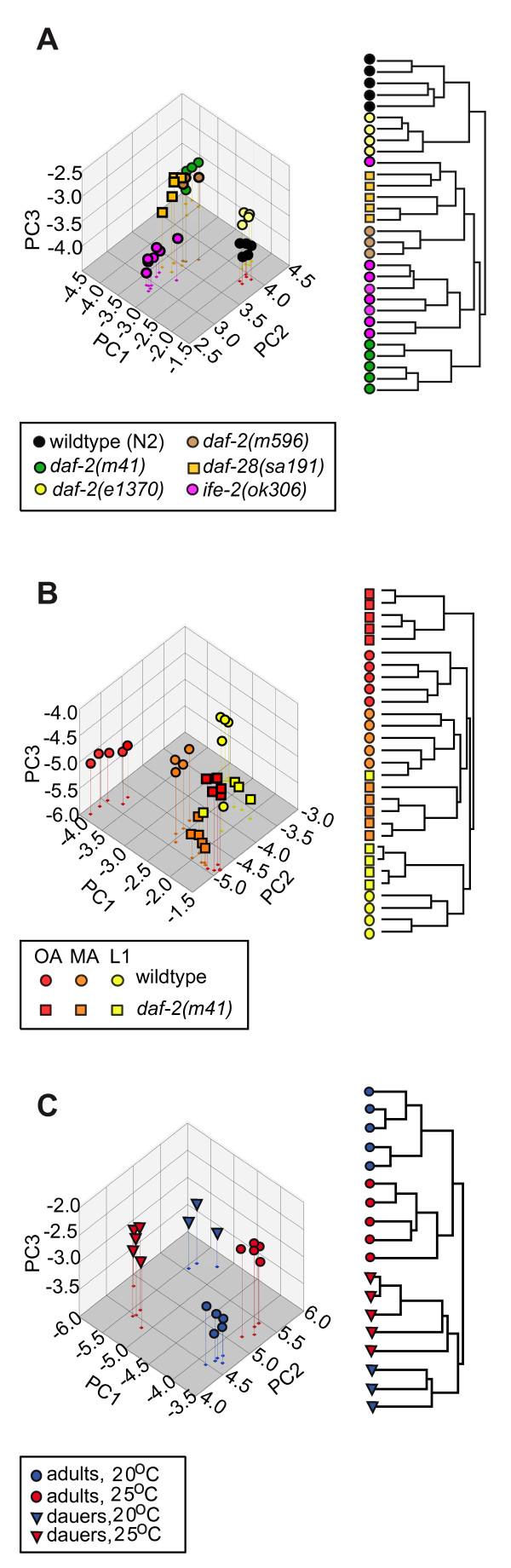
**Longevity mutants and dauer larvae have distinctive metabolic profiles**. **A**. PCA of four IIS mutants and a long-lived translation-defective mutant, *ife-2(ok306)*; cluster analysis separates the mutants into distinct groups. *daf-2(e1370) *is the most similar to wild-type. *ife-2(ok306)*, while distinctive relative to wild-type and all other mutants, is not distinctive relative to the IIS mutants as a class. In this experiment mutant and wild-type worms were initially raised at 15°C, transferred to 25°C at L4, and assayed at 240 hours post bleaching. The first three PCs respectively account for 40, 20 and 16% of the variance. **B**. PCA shows that *daf-2(m41) *has a distinctive profile even at L1 (15 hours); and appears to become successively more distinctive as a middle-aged adult (144 hours) and old (240 hours) adult. The first three PCs explain, respectively, 35, 26 and 11% of the variance. **C**. PCA of dauers and wild-type worms (240 hours) raised at two temperatures, 20°C and 25°C. The PCA and cluster analysis shows that dauers and adults clearly have distinct metabolomes as do worms raised at 20°C rather than 25°C. The first three PCs explain, respectively, 48, 16 and 11% of the variance, with PC1 distinguishing stages and PCs 2 and 3 distinguishing temperature. Note that the relative position of the samples raised at the two temperatures is reversed along PC2, a consequence of strong temperature × stage interaction.

Many IIS mutations exist and they differ in the severity and kind of their phenotypic effects [3,34]. So, in a separate experiment we simultaneously studied three *daf-2 *mutations: *m41, e1370 *and *m596 *as well as *daf-28(sa191) *which disrupts an insulin-like ligand thought to bind DAF-2. DAF-28 is thought to activate DAF-2 and so promote normal, reproductive growth and longevity, but *sa191 *is a dominant negative gain-of-function allele [35]. Like *daf-2 *hypomorphs, *daf-28(sa191) *is partly dauer constitutive, has long-lived adults, and can be repressed by mutations in *daf-16 *[35]. In this experiment, we used fewer samples of worms per genotype than in previous experiments, but sampled three ages, L1 (15 hours), middle-age (144 hours) and old age (240 hours), and raised them first at 15°C and then at 25°C. Considering just the old-age samples we found that all of these mutants have distinctive metabolic profiles, with *e1370 *and *m41 *having respectively the least and most distinctive metabolic phenotypes relative to wild-type (Figure 2A). The rank order of allele strength for longevity at 22.5°C and 25°C is *e1370 *<*m41 *<*m596 *(our data not shown; [3]), but *daf-2 *alleles have a variety of phenotypic effects which do not all show the same rank order of severity [3,34,39]. The three *daf-2 *mutations affect different parts of the receptor: *e1370 *disrupts the intracellular tyrosine kinase domain while *m41 *and *m596 *disrupt, respectively, the Cysteine Rich and Leucine Rich L2 extracellular domains [34,39]. Some L2 domain mutations in the human Insulin receptors have very low ligand binding affinity [40]; a similar property of *daf-2(m596) *may explain why its metabolome resembles that of *daf-28(sa191) *which disrupts a putative ligand.

When surveying the four *daf-2(-) *mutants, we also looked at another kind of long-lived mutant, *ife-2(ok306)*, which disrupts a gene encoding an isoform of the eukaryotic translation initiation factor, eIF4E [9]. Since this mutation does not require DAF-16 to confer increased longevity, it is thought that IFE-2 works either downstream or in parallel to DAF-2 to regulate longevity. We found that the metabolic profile of *ife-2(ok306) *is very similar to that of the ILS mutants: cluster analysis and PCA do not clearly separate *ife-2(ok306) *worms from ILS mutants (Figure 2A).

Since we sampled all of our mutants at three ages we were also able to study, at least crudely, when the mutant worms acquired their distinctive metabolic profiles. PCA and cluster analysis shows that all mutants had distinctive metabolic profiles even as larvae, but in all cases the metabolic profiles became increasingly different from wild-type with age (Figure 2B). Since reproduction has not yet begun in L1 larvae, which do not even have gonads, the distinctive profiles of the long-lived mutants cannot be entirely due to a decrease in metabolic resources allocated to reproduction.

Finally, in this same experiment, we also studied dauer larvae. Dauers form when L2 worms are crowded or deprived of food. They do not feed, have very distinctive transcriptional profiles, physiologies and morphologies, are very stress resistant and also do not age [1]. We raised dauers at two temperatures, 20°C (n = 3) and 25°C (n = 5), and compared them to old adults (240 hours) raised at the same temperatures (n = 5 for both). Clustering and PCA showed that dauers and adults have unambiguously distinct metabolic profiles as do worms raised at different temperatures, with temperature nested within the two stages (Figure 2C). Comparing dauers to L1s or young adults gave very similar results (data not shown). We found that metabolite levels showed strong stage × temperature interactions. This is reflected in the reversal of the relative positions of dauers and adults along the PC 2 axis depending on the temperature and also in analysis of variance on individual bins (data not shown).

### The metabolic signature of long life in worms

What are the metabolic features of long-life? In *C. elegans*, many mutants and environmental treatments confer increased longevity, but the devices by which they do so, or whether or not they are the same, remains unclear. One reason for this is that few studies assay more than one class of long-lived worm using the same technology and experimental conditions. Since we have studied three classes of long-lived worm, dauers, IIS defective and a translation-defective mutant, using the same metabolic profiling technique, we can directly compare them and ask what, if anything, do they have in common?

**Figure 3:**
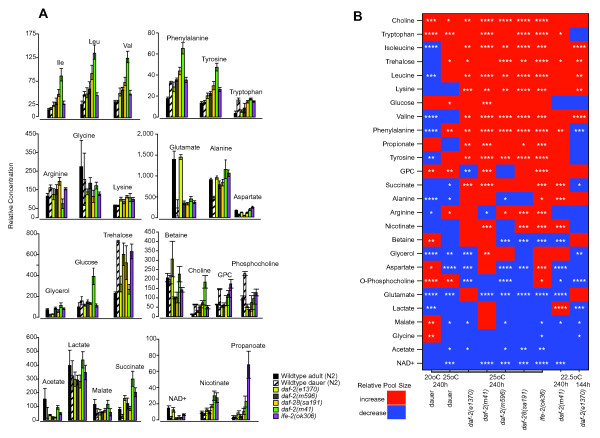
**The metabolic signature of long-life**. **A**. Relative concentrations of 26 metabolites in worms sampled at 25°C and 240 hours post-hatching. **B**. Summary of metabolic responses. Here we show, for all experiments, the observed response in long-lived worms relative to appropriate controls and rank them by their consistency; *p *values for individual experiment are given in each cell: * *P *< 0.05; ** <0.01; *** <0.001; **** <0.0001. For all data and statistics see Additional file 1.

To do this we determined the relative concentrations of 26 metabolites (Figure 3A; Additional file 1). Then, to identify the metabolic signatures of long-life we ranked them by consistency and direction of response in long-lived worms relative to wild-type (Figure 3B). Although this figure shows the results of all of our experiments, here we focus on the single experiment in which worms were raised at 25°C and sampled at 10 days after hatching.

We found that the metabolic responses of our long-lived worms were strikingly similar. More than half of the examined metabolites show qualitatively similar changes in dauers, IIS mutants, and *ife-2 *mutant worms. This result was surprising since IIS and *ife-2 *mutations ostensibly influence very different aspects of the worm's physiology. We propose that these metabolites constitute a minimal metabolic signature of long-life in worms.

One of the signature metabolites was the disaccharide trehalose. An important carbohydrate storage molecule in nematodes, trehalose is thought to confer stress resistance in many invertebrates [41-43]. Previous studies have shown that the expression of genes involved in its synthesis are elevated in dauers and IIS mutants so our finding that trehalose pool sizes are elevated in dauers and IIS mutants was expected; indeed, trehalose has been proposed as a *longevity assurance sugar *[19,44]. *ife-2(ok306) *worms show high trehalose levels as well implying that a deficiency in protein synthesis can affect carbohydrate metabolism as well.

This similarity across different classes of long-lived worms is also seen in amino acid levels. Of the 12 amino acids we studied, 11 are regulated in IIS mutants. Of these, 10 are regulated in the same way in *ife-2(oK306) *and 5 are in dauers. We also found the following metabolites consistently regulated across dauer, IIS, and *ife-2(0) *mutants: choline, phosphocholine, and glycerophosphocholine (GPC), which are associated with lipid metabolism; acetate, malate and succinate, which are associated with carbohydrate metabolism; propanoate and NAD^+^. Some of these longevity-signature metabolites are expected from previous studies of long-lived worms. This is particularly true of metabolites that have a role in carbohydrate metabolism and we consider them in greater detail below. Others, such as altered pool sizes of amino acids, choline, phosphocholine, propanoate and NAD^+^, could point to new mechanisms of longevity assurance in *C. elegans*. We also note that, although we have ignored metabolic responses peculiar to particular worm strains, we cannot exclude the possibility that they influence longevity as well.

### Autophagy and the dauer metabolome

Although the dauer metabolome resembles that of long-lived mutants in many ways, we also found that it has some unique features. We detected pools of two post-translationally modified amino acids, phosphoserine and hydroxyproline, in dauers and only dauers. Since hydroxylation and phosphorylation generally take place on peptides rather than free amino acids, these pools are likely the result of protein degradation. The most obvious source of free hydroxyproline is collagen: the *C. elegans *genome contains approximately 175 genes encoding collagens which are used in the basement membranes and cuticle, two prolyl 4-hydroxylases, and at least five peptidases that are required for the processing or turn-over of cuticle collagens [45,46]. The source of free phosphoserine is less obvious, however, given that we studied high concentration metabolites, they probably are not derived from the phosphorylated serines found in signalling pathways but rather represent structural components. We suspect that the phosphoserine pool seen in dauers is derived from serine phosphoglyceride lipids freed by turnover of membrane phospholipids. This is supported by the observation that choline compounds (choline, phosphocholine, and GPC) are also strongly increased in dauers, and altered choline compound concentrations are frequently observed in mammalian tumours where they mark the membrane turnover characteristic of rapidly proliferating cells [47]. Since dauers do not feed they rely on energy stores such as fats and glycogen to survive, and much evidence shows that the beta-oxidation and glyoxylate pathways that metabolize fatty acids are upregulated in dauers [19,48-50]. One explanation, then, for the elevated levels of these modified amino acids is that dauers are utilizing spare extracellular matrix, and other proteins, as yet another energy store. Consistent with this idea, dauer morphogenesis requires extensive autophagy [51]. A mutation that abolishes autophagy also decreases longevity specifically in *daf-2(e1370) *which implies that high levels of autophagy promote long-life [51]; if so we did not detect any obvious signatures of this.

### Some, but not all, signature metabolite responses require DAF-16

Much evidence shows that the longevity prolonging effects of IIS mutants are mediated by the FOXO transcription factor, DAF-16 [2,52,53]. Down-regulation of DAF-2 signalling results in nuclear localization and hence activation of DAF-16 which, in turn, activates or represses many genes which contribute to longevity [14,54,55]. Since the longevity prolonging effect of *daf-2 *mutants is repressed by null mutations of *daf-16*, one way of disentangling the phenotypes of IIS mutants that contribute causally to longevity from those that do not, is to ask whether they, too, depend on DAF-16 activity. The reasoning, previously applied to transcriptomic and proteomic data, is that any molecular phenotype that contributes to longevity should be abolished by inhibiting DAF-16 activity just as longevity itself [15,21].

**Figure 4:**
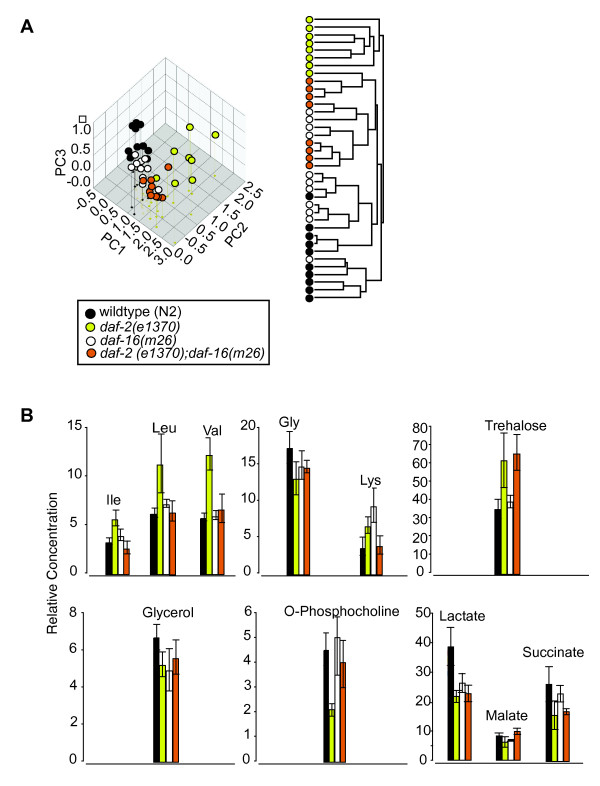
**DAF-16 dependence of metabolites**. **A**. PCA of metabolic profiles of wild-type, single mutant *daf-2(e1370) *and *daf-16(m26) *and double-mutant *daf-2(e1370);daf-16(m26) *samples. PCA and cluster analysis shows that *daf-2(e1370) *have the most divergent metabolism of the four genotypes, implying that some of the distinctive features of the *daf-2 *metabolome is *daf-16 *dependent. The first three PCs explain, respectively, 31, 21 and 13% of the variance; most of the separation between *daf-2 *and other genotypes is along PC2. In this experiment mutant and wildype worms were initially raised at 15°C, transferred to 22.5°C at L4, and assayed at 144 hours post bleaching. **B**. Relative concentrations of 11 metabolites in worms sampled at 22.5°C and 144 hours post hatch. Four metabolites - isoleucine, valine, leucine and phosphocholine - show classical DAF-16 dependence, the rest show more complex patterns of epistasis or none.

To find out whether IIS control over metabolism was also DAF-16 dependent we compared the metabolomes of 144 hour-old wild-type worms to worms carrying either another *daf-2 *allele, *e1370*, or the null *daf-16*(*m26*) mutation, or both. Unsupervised methods (PCA and HCA) divide these samples into two major groups: a cluster which contains 7/8 *daf-2(-) *samples and a cluster that contains the rest (Figure 4A). Thus the metabolic phenotype of *daf-2 *is at least partially DAF-16 dependent; however the double mutants form a sub-cluster clearly distinct from wild-type implying that not all of the *daf-2 *phenotype is so.

By measuring metabolite levels directly in single mutant *daf-2(e1370) *and *daf-16(m26) *and double mutant *daf-2(e1370);daf-16(m26) *worms we were able to apply this test to 11 metabolites (Figure 4B). Of these only four, phosphocholine, and the closely related amino acids isoleucine, valine, and leucine, showed the classic pattern of DAF-16 dependence: concentrations of each were substantially elevated or repressed in *daf-2(-)*, but not *daf-16(0) *or double mutant samples. Several other metabolites showed more complex patterns of epistasis. For example, lysine, lactate and glycerol concentrations were all significantly different in either *daf-16(0) *or *daf-2(-);daf-16(0) *worms or both compared to wild-type worms (*P = *0.05; two-tailed t-test) and so fail the classical test of DAF-16 dependence. Surprisingly, trehalose showed no sign of DAF-16 dependence: *daf-16(0) *samples have wild-type levels of the sugar, but double mutants are not different from *daf-2(-)*. This is in contrast to previous results shown by transcriptomic studies [15,19], which may perhaps reflect the fact that changes in gene expression levels alone do not necessarily equate to functional differences [56,57].

Classical DAF-16 dependence make isoleucine, valine, leucine and phosphocholine strong candidates for having a causal role in long life, particularly as all four are signature metabolites as defined above. Conversely, the absence of DAF-16 dependence in trehalose suggests that DAF-2 regulates it via another transcription factor parallel to DAF-16 and that it may not contribute to long life. The interpretation of non-classical *daf-16 *epistasis is less clear. The classical test supposes that DAF-16 is fully repressed in normal worms, but activated in the absence of DAF-2 signalling by translocation of the transcription factor from cytoplasm to nuclei [55,58]. This model is certainly too simple since *daf-16(0) *mutants have a variety of subtle phenotypes such as rapid growth, early reproduction and a slightly reduced lifespan, and normal worms have at least some DAF-16 visible in their nuclei [59,60]. Some metabolites are, then, also apparently sensitive to low levels of DAF-16 activity.

### Metabolic targets of DAF-2 signalling

We have shown that the pool sizes of many metabolites differ between long-lived and normal worms and that some of these differences are shared by various long-lived mutants, in particular the several *daf-2 *alleles that we studied. But what genes does *daf-2 *regulate that result in these changes? In order to investigate this, we mapped some of our signature metabolites onto a standard metazoan metabolic network so that we could identify those parts of the network that are altered in *daf-2 *worms. We then asked whether the genes encoding metabolic enzymes that work in the same parts of the network are regulated as well, and if so, whether the two sources of data could be used to give an economical account of how the metabolism of *C. elegans *is altered in *daf-2 *worms. As an initial guide we used NEMAPATH [61] to identify, for each metabolite, the pathways in which they might work and the *C. elegans *genes that might act in them. We then interrogated a previously published global expression dataset based on *daf-2(e1370) *worms raised under conditions similar to ours [20] and examined the expression patterns of these genes for patterns of co-regulation.

**Figure 5:**
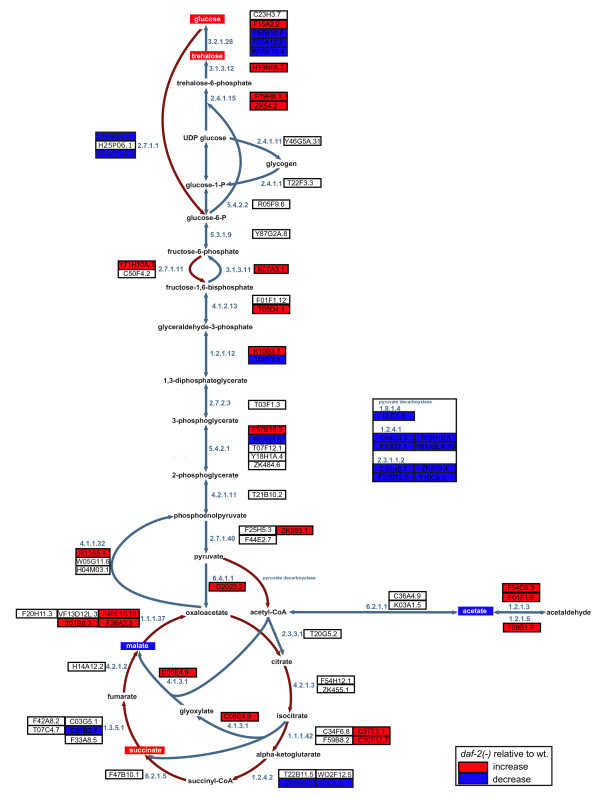
**Carbohydrate metabolism in *daf-2 *worms**. Five signature metabolites - malate, acetate, succinate, glucose and trehalose - are linked by three major pathways: the glyoxylate shunt, gluconeogenesis, and starch metabolism. Expression data [20] shows that these three pathways (blue lines) are upregulated in *daf-2(e1370) *worms. Glycolysis and citric acid cycle genes (brown lines) are, by contrast, generally downregulated or unregulated. In this model, carbon from acetate or fatty acid metabolism enters the glyoxylate pathway mediated by increased expression isocitrate lyase (4.1.3.1) and malate synthase (4.1.3.2) which are encoded by a single gene, *gei-7*. The product of this pathway, malate, is then converted to oxaloacetate by cytosolic malate dehydrogenase (1.1.1.37) which then results, via gluconeogenesis, to the production of carbohydrates. The irreversible steps of gluconeogenesis are catalysed by phosphoenolpyruvate carboxykinase (4.1.1.32, PEPCK), pyruvate carboxylase (6.4.1.1), and fructose 1,6-biphosphatase (3.1.3.11). Most of the genes encoding components of these enzymes are upregulated in *daf-2(-) *worms. In most animals, glucose is synthesized from glucose-6-phosphate by glucose-6-phosphatase (3.1.3.9) but *C. elegans *does not contain a homologue of this gene. We suppose, then, that glucose is produced by the metabolism of trehalose by trehalase (3.2.1.28). Several trehalase genes are downregulated in *daf-2 *worms implying a reduced flux to glucose. However, glucose demand is also probably reduced since two genes encoding hexokinase (2.7.1.1), responsible for a irreversible reaction in glycolysis, are repressed in *daf-2 *worms as are many of the genes encoding the pyruvate dehydrogenase complex that links glycolysis to the citric acid cycle via acetyl-CoA. In contrast to the glyoxylate pathway genes, TCA genes are not generally regulated in *daf-2(-) *worms. Genes and metabolites are taken to be regulated if *P = *0.05 (Additional file 2).

We found that at least five of the metabolites regulated in *daf-2(-) *worms, malate, acetate, succinate, glucose and trehalose, were linked by three major pathways: the glyoxylate shunt, gluconeogenesis, and starch metabolism. Consistent with previous studies [15,19,21] we found that genes encoding enzymes in these pathways are up-regulated in *daf-2(-) *worms. In all, the *C. elegans *genome contains 38 genes encoding components of 20 enzymes that work in these pathways. Of these 38 genes, 14 are significantly up-regulated, 5 are significantly down-regulated, 18 are not regulated, and 1 has no data (Figure 5, Additional file 2).

While the observed shifts in carbohydrate metabolism might have been expected from the results of gene expression studies, the changes in amino acid metabolism shown by our data were not. Most amino acid pools are upregulated in long-lived worms (8/12 in the IIS mutants; 10/12 in *ife-2*). One possible explanation for this is that protein synthesis is generally repressed in IIS mutants as it is in *ife-2 *[9] and that the amino acid pool represents a surplus. *daf-2(e1370) *worms may have reduced protein synthesis since the expression of their t-RNA synthetases are generally repressed (Additional file 2). However, there is no correlation between amino acid pool size and tRNA synthetase expression (data not shown). It is more likely then that amino acid pool sizes are dictated by catabolic pathways that direct them to energy production or other uses. Consistent with this, many genes that encode components of phenylalanine and tyrosine catabolic pathways, among them tyrosinase and phenylalanine hydroxylase, are regulated in *daf-2 *(Additional file 2) [21,62]. Intriguingly, melanin, a tyrosine metabolite, has recently been discovered in worm cuticles where it is thought to have a protective function [63].

The most striking response among the amino acids, however, is the upregulation of the branched chain amino-acids (BCAAs) isoleucine, leucine and valine. The pool sizes of these amino acids are positively correlated across long-lived mutants (Figure 3A). Furthermore, unlike most other metabolites, their upregulation in *daf-2 *is entirely DAF-16 dependent, making them strong candidates for being causally involved in longevity (Figure 4B). Like other animals, *C. elegans *cannot synthesize these amino acids [64], and so any difference in their relative concentrations must be due to a change in either protein turnover or their catabolism. In fact, BCAA pool sizes are co-regulated in many circumstances such as growth in worms [31] or obesity in humans [65,66]. This co-regulation is a consequence of them sharing the first two steps in their catabolic pathways: transamination by BCAT and oxidative carboxylation by the mitochondrial BCKD enzyme complex [65]. In *daf-2(e1370) *worms, BCAT expression is wild-type, but all four genes encoding components of the BCKD complex are strongly downregulated (Additional file 2). We hypothesize that downregulation of the BCKD complex is responsible for the increased BCAA pool sizes of *daf-2 *worms. This hypothesis also suggests a way to manipulate BCAA pool sizes to test their contribution to long life. Strong inactivation of BCKD-complex genes in worms causes severe embryonic and larval phenotypes ([67]; wormbase.org) and, in humans, maple syrup urine disease, a metabolic disorder resulting in encephalopathy and death [68]; however, it remains possible that more subtle elevation of BCAA levels by diet or partial downregulation of the BCKD complex will confer long life.

## Conclusions

By studying the metabolic profiles of a range of long-lived worms we have identified a metabolic signature of long life common to dauers, IIS mutants and a translation defective mutant. Some of the metabolites that comprise this signature, such as those involved in carbohydrate metabolism, are expected from studies of global gene expression; others, such as those involving amino acid metabolism, are new. The existence of a common metabolic signature for long life suggests that longevity pathways that have been previously considered independent may, in fact, regulate the same regions of the metabolic network. By interrogating an existing global gene expression profile dataset on *daf-2 *worms, we have identified some of those regions. We find that the changes in carbohydrate metabolism can be explained by upregulation of the glyoxylate shunt and gluconeogenesis. We also find that the general elevation of amino acid pool sizes in long-lived worms is likely due to regulation of catabolic pathways that divert amino acids away from protein synthesis and to energy metabolism or other survival functions. Dauers show many of the features of long-lived mutants, but they also show elevated levels of phosphoserine, hydroxyproline, and choline compounds; we suggest that these are probably the consequence of extensive autophagy.

Although we have shown how the study of metabolite concentrations and gene expression can be used together to give a consistent account of the metabolism of long-lived worms, we recognize that we have considered only a few of the many metabolic differences that may give these worms their remarkable life-spans. Ultimately, we would like to provide a general metabolic model for long-life - one that quantitatively explains longevity in terms of the expression of genes that encode metabolic enzymes, the activity of those enzymes, and the metabolic concentrations and fluxes that result from them. That goal however, requires a well-founded native metabolic network for *C. elegans *as well as a general account of its kinetics and how they are regulated, none of which currently exist. Nevertheless, the recent development of system-wide metabolic models, particularly of microbes, suggests that such a global model of *C. elegans *metabolism may be possible in the near future [69-72]. Our study begins to provide the empirical basis upon which it will depend.

## Methods

### Strains

We used the following strains: wild-type N2 Bristol, DR26 *daf-16(m26)*, CB1370 *daf-2(e1370)*, DR1564 *daf-2(m41)*, DR1565 *daf-2(m596)*, DR1309 *daf-16;daf-2(m26;e1370)*, JT191 *daf-28(sa191)*, KX15 *ife-2(ok306) *and RB579 *ife-2(ok306)*, the outcrossed version of KX15. They were provided by the *Caenorhabditis elegans *Center (CGC) at the University of Minnesota.

### Worm culture

Worms were grown using standard techniques. *daf-2(-) *alleles are temperature-sensitive dauer constitutive. Synchronized populations were grown at the permissive temperature, 20°C, until L4 and then transferred to 22.5°C or 25°C. Controls and non-ts strains were treated in the same way. Dauers were induced by growing worms as above but at high density. Worms were sampled for NMR at either 15 h (L1), 144 h (adult, six days), or 240 h (adult, 10 days) post hatch. To ensure that only old worms were sampled at 10 days, their offspring were removed by repeated filtering using 50 μm Nitex membranes (Sefar Ltd., Bury, UK).

### Nuclear magnetic resonance (NMR) spectroscopy

For NMR analysis worms were washed off plates and collected in 0.5 ml of M9 buffer [73], quick-frozen in liquid nitrogen and stored at -80°C. We then ground the tissue at liquid nitrogen temperatures in a mortar and pestle, and added 2 ml ice-cold methanol directly to the ground tissue to give a final concentration of 80% methanol. We transferred the extract to microcentrifuge tubes, and rinsed the mortars with an additional 2 ml of 80% methanol. We then centrifuged the extracts (10 minutes, 16,000 g) and dried the supernatants in a rotary vacuum concentrator. We rehydrated each sample in 650 μl of NMR buffer (100% ^2^H_2_O, 0.97 mM sodium trimethylsilyl-2,2,3,3-^2^H_4_-propionate (TSP), 0.1 M phosphate buffer pH 7.0), centrifuged again to remove any particulate matter, and transferred 600 μl to 5 mm NMR tubes.

NMR spectra were acquired essentially as described by Beckonert et al. [74] using a Bruker Avance DRX600 spectrometer (Bruker BioSpin, Rheinstetten, Germany) with a field strength of 14.1 T and consequent 600 MHz ^1^H resonance frequency, equipped with a 5 mm cryogenically-cooled inverse geometry probe. A 1D NOESY pulse sequence was used for water suppression, with an acquisition time of 1.36 s, and an additional relaxation delay of 3.5 s, with presaturation during the relaxation recovery and 0.1 s mixing time giving a 5 s recycle time; we collected 160 transients per sample, following four dummy scans to allow the system to approach a steady state. The data were acquired into 32 K points over a 12 kHz spectral width.

### NMR data processing and analysis

We carried out initial data processing in iNMR v.2. The summed transients were multiplied by an exponential apodization function equivalent to 0.5 Hz line broadening and zero-filled by 50%, followed by Fourier transformation. The spectra were referenced to the TSP resonance at 0 ppm, and phase correction and first-order baseline correction carried out using the software's proprietary algorithms. We visually identified peaks in the spectra and divided them manually into bins (integrals); compared to equal-interval binning of the entire spectrum, this has the effect of reducing the total number of variables, aligning each bin more closely with an individual resonance, and excluding spectral regions that contain only noise across all samples. Around 40 detectable metabolites could be readily identified in routine 1D spectra of the worm extracts (Additional file 3). There were also a number of resonances from as-yet unassigned metabolites (for example, singlets at δ 1.993 and δ 1.986 ppm represent probable *N*-acetyl groups). In addition, we re-processed all spectra in Chenomx NMR Suite 4.6 (Chenomx, Edmonton, AB, Canada) and quantified metabolite concentrations for selected metabolites by computer-assisted manual fitting of metabolites. This software fits idealized spectra made up of combinations of Lorentzian peaks, based on authentic standards [75]. We assigned metabolite resonances by comparing their multiplicity and chemical shift to compounds found in the Chenomx database. This was supplemented by 2D NMR experiments (COSY and HSQC) acquired for typical samples, and additional comparisons to our own in-house standards data and other online databases. All of the metabolites fitted were present in the Chenomx proprietary database, except for trehalose, which we added to the database.

We then normalized the data by dividing each profile by a single normalization factor, the median fold change across all compounds relative to a reference profile (a median of all profiles), as described by Dieterle et al. [76], and log-transformed them by log_10_(n_i _+ x). The transformation reduced the dominating effect of the high intensities of a few metabolites, so that intensity of otherwise weak and insignificant peaks is increased. The constant x was chosen such that the dependence between standard deviation and intensity was removed for a series of technical replicates, that is, increasing homoscedasticity (the principle is discussed by Purohit et al. [77], although a different transform was used by these authors). Multivariate analyses (principal components analysis, PCA, and hierarchical cluster analysis, HCA) were performed in either Aabel (Gigawiz, Tulsa, OK, USA) or JMP (SAS UK, Marlow, UK) as appropriate. Using the fitted data (that is, from Chenomx), we then tested the level of each metabolite in each experiment against its appropriate control (Additional file 1). In order to identify those metabolites that are generally regulated across multiple longevity treatments, we used a Fisher's combined probability test (Additional file 1, Part 4). As an additional test of the overall consistency in the pattern of regulation, we applied a sample randomization test and found as a whole the data set is highly structured (*P *< 1e-10).

### Gene expression analysis

We downloaded Shaw et al.'s [20] gene expression data from PUMAdb http://puma.princeton.edu/, and interrogated it for the expression of genes putatively encoding all metabolic enzymes. In these experiments, *daf-2(e1370) *worms were grown at 25°C and so the results are also relevant to our data. Gene identities and metabolic pathways were based on NemaPath [61], a version of KEGG [78-80] native for nematode sequences http://www.nematode.net/cgi-bin/keggview.cgi.

## Abbreviations

BCAA: branched chain amino acid; BCKD: branched chain ketoacid dehydrogenase; COSY: correlated spectroscopy; GPC: glycerophosphocholine; HCA: hierarchical cluster analysis; HSQC: heteronuclear single quantum coherence; IIS: insulin/insulin-like signalling; NMR: nuclear magnetic resonance; NOESY: nuclear Overhauser effect spectroscopy; PCA: principal component analysis; TSP: sodium trimethylsilyl-2,2,3,3-^2^H_4_-propionate.

## Authors' contributions

SF and SKD carried out metabolomic studies and primary analysis of data and helped write the paper. JMV and JSS carried out further analyses. JGB and AML conceived and coordinated the study, constructed the figures, and wrote the paper. All authors read and approved the final manuscript.

## Supplementary Material

Additional file 1 **Table S1**. Metabolite concentrations in long-lived and normal worms based on manual computer-aided fitting to 1H NMR spectra (see Materials and Methods for further details). Click here for file

Additional file 2 **Table S2**. Expression of genes encoding enzymes in selected metabolic pathways. Relative expression in *daf-2(e1370) *adults compared to wild-type worms; data from reference [20]. Click here for file

Additional file 3 **Table S3**. List of assigned metabolites detected in worm samples by 1D NMR, and associated resonance chemical shifts and multiplicity. Only the most characteristic resonances are listed in the table. Click here for file
