# Supplementary material for: A metabolic signature of long life in Caenorhabditis elegans
Source: BMC Biol. 2010 Feb 10;8:14. doi: 10.1186/1741-7007-8-14 (PMC2829508; doi:10.1186/1741-7007-8-14)
Supplement: Additional file 1 — Table S1. Metabolite concentrations in long-lived and normal worms based on manual computer-aided fitting to 1H NMR spectra (see Materials and Methods for further details). [file 1741-7007-8-14-S1.PDF]

SI Table 1, part 1

Experiment 1
Comparison N2 (wildtype) v. *daf-2(m41)*, 22.5oC, adult 240h
Data Relative concentrations determined from NMR spectra and computer-assisted manual fitting of metabolites
Analysis t-test

	**Genotype**	N2	*daf-2(m41)*		
	**Statistic**	Mean ± 95%CI	Mean ± 95%CI	% change	P, t-test
**Compound Class**	**Compound**				
Amino Acid	Alanine	248.43 ± 28.81	329.39 ± 27.19	133	7.0E-04
Amino Acid	Arginine	88.59 ± 16.03	80.8 ± 9.36	91	4.2E-01
Amino Acid	Aspartate	59.88 ± 5.89	28.71 ± 5.07	48	1.5E-07
Amino Acid	Glutamate	710.77 ± 105.48	362.93 ± 64.15	51	2.1E-05
Amino Acid	Glycine	74.39 ± 9.56	63.85 ± 6.88	86	9.5E-02
Amino Acid	Hydroxyproline	-	-	-	-
Amino Acid	Isoleucine	36.23 ± 5.88	39.52 ± 3.31	109	3.5E-01
Amino Acid	Leucine	64.89 ± 8.95	67.68 ± 3.12	104	5.7E-01
Amino Acid	Lysine	52.23 ± 9.87	54.39 ± 7.73	104	7.4E-01
Amino Acid	Phenylalanine	22.15 ± 1.5	26.04 ± 1.83	118	4.3E-03
Amino Acid	Phosphoserine	-	-	-	-
Amino Acid	Tryptophan	6.11 ± 1.3	9.03 ± 1.73	148	1.6E-02
Amino Acid	Tyrosine	12.85 ± 1.35	13.02 ± 1.06	101	8.5E-01
Amino Acid	Valine	53.17 ± 9.3	63.27 ± 5.43	119	8.1E-02
Organic Acid	Acetate	31.97 ± 6.2	30.21 ± 5.23	95	6.7E-01
Organic Acid	Lactate	85.15 ± 15.78	174.57 ± 17.24	205	3.1E-07
Organic Acid	Malate	29.34 ± 6.22	21.21 ± 2.57	72	2.8E-02
Organic Acid	Nicotinate	3.06 ± 0.94	5.67 ± 0.63	185	2.2E-04
Organic Acid	Propanoate	11.22 ± 2.86	13.8 ± 3.37	123	2.7E-01
Organic Acid	Succinate	83.69 ± 18.17	138.95 ± 14.08	166	1.3E-04
Nucleotide	NAD+	4.99 ± 1.35	1.97 ± 0.55	40	6.4E-04
Carbohydrate	Glucose	37.48 ± 8.01	30.7 ± 3.09	82	1.4E-01
Carbohydrate	Trehalose	35.64 ± 6.62	46.58 ± 5.11	131	1.8E-02
Phospholipid-related	Betaine	90.92 ± 10.81	54.55 ± 10.3	60	1.2E-04
Phospholipid-related	Choline	12.93 ± 2.85	17.66 ± 3.4	137	5.0E-02
Phospholipid-related	GPC	14.58 ± 2.92	15.62 ± 3.07	107	6.4E-01
Phospholipid-related	Glycerol	28.66 ± 8.66	29.44 ± 6.98	103	8.9E-01
Phospholipid-related	O-Phosphocholine	16.58 ± 3.74	11.39 ± 1.71	69	2.3E-02

SI Table 1, part 2

Experiment 2
Comparison N2: old adult (240h) v. dauer, 20.0oC
Data Relative concentrations determined from NMR spectra and computer-assisted manual fitting of metabolites
Analysis t-test

	**Stage/Age**	adult 240h	dauer	dauer	dauer
	**Statistic**	Mean ± 95%CI	Mean ± 95%CI	% change	t-test
**Compound Class**	**Compound**				
Amino Acid	Alanine	1383.48 ± 30.91	548.59 ± 172.02	40	1.5E-05
Amino Acid	Arginine	221.38 ± 13.61	141.38 ± 55.53	64	1.8E-02
Amino Acid	Aspartate	78.4 ± 5.33	104.42 ± 19.84	133	2.8E-02
Amino Acid	Glutamate	749.76 ± 27.84	340.67 ± 77.49	45	1.4E-05
Amino Acid	Glycine	141.99 ± 10.3	211.17 ± 25.49	149	1.0E-03
Amino Acid	Hydroxyproline	-	85.3 ± 13	-	-
Amino Acid	Isoleucine	21.06 ± 2.37	8.08 ± 1.33	38	5.4E-05
Amino Acid	Leucine	32.71 ± 1.85	17.89 ± 5.08	55	6.1E-04
Amino Acid	Lysine	70.32 ± 9.26	57.77 ± 13.77	82	1.7E-01
Amino Acid	Phenylalanine	30.3 ± 1.52	16.83 ± 3.12	56	8.1E-05
Amino Acid	Phosphoserine	-	169 ± 31	-	-
Amino Acid	Tryptophan	7.44 ± 1.13	22.64 ± 2.18	304	3.8E-06
Amino Acid	Tyrosine	15.96 ± 1.27	10.14 ± 2.55	64	3.6E-03
Amino Acid	Valine	46.86 ± 1.11	20.13 ± 4.22	43	3.0E-06
Organic Acid	Acetate	56.56 ± 6.4	49.69 ± 9.61	88	2.7E-01
Organic Acid	Lactate	582.81 ± 52.8	305.45 ± 57.02	52	2.2E-04
Organic Acid	Malate	67.11 ± 14.93	106.42 ± 12.1	159	6.2E-03
Organic Acid	Nicotinate	7.79 ± 2.01	6.3 ± 1.68	81	3.2E-01
Organic Acid	Propanoate	18.15 ± 3.7	32.58 ± 21.02	179	1.8E-01
Organic Acid	Succinate	130.66 ± 12.41	80.21 ± 57.15	61	1.0E-01
Nucleotide	NAD+	12.69 ± 3.2	10.73 ± 3.86	85	4.7E-01
Carbohydrate	Glucose	77.42 ± 5.66	147.72 ± 129.56	191	2.7E-01
Carbohydrate	Trehalose	425.26 ± 19.58	420.66 ± 182.25	99	9.6E-01
Phospholipid-related	Betaine	124.62 ± 4.66	238.67 ± 52.71	192	2.0E-03
Phospholipid-related	Choline	9.95 ± 5.24	59.44 ± 13.4	597	1.6E-04
Phospholipid-related	GPC	70.22 ± 4.99	178.24 ± 47.95	254	1.6E-03
Phospholipid-related	Glycerol	105.71 ± 10.05	21.63 ± 13.32	20	2.0E-05
Phospholipid-related	O-Phosphocholine	57.5 ± 2.55	499.49 ± 68.52	869	1.9E-06

SI Table 1, part 3

Experiment 2
Comparison N2: Larval (L1, 15h), middle-aged adult (144h) or old adult (240h) v. dauer, 25.0oC
Data Relative concentrations determined from NMR spectra and computer-assisted manual fitting of metabolites
Analysis (1) a t-test of each reproductive stage v. dauer; (2) a t-test of the combined reproductive stages v. dauer; (3) a 2-way ANOVA with interaction for only the 240h data: temperature, stage and interaction as effects

**Compound Class**	**Stage/Age**<br>**Statistic**<br>**Compound**	L1 15h	adult 44h	adult 240h<br>Mean ± 95%CI	dauer<br>Mean ± 95%CI	dauer/15h<br>% change	dauer v.15h<br>t-test	dauer/144h<br>% change	dauer v. 144h<br>t-test	dauer/240h<br>% change	lauer v.240h<br>t-test	dauer/all<br>% change	dauer v. all<br>t-test	Two-way ANOVA on dauers<br>P,F-test	P,F-test	P,F-test
Amino Acid	Alanine	1235.36 ± 508.62	1923.87 ± 705.61	879.64 ± 32.59	456.41 ± 200.89	37	0.050	24	2.2E-02	52	1.4E-02	35	6.9E-04	1.0E-04	4.0E-04	7.5E-03
Amino Acid	Arginine	195.69 ± 150.14	387.69 ± 83.43	116.37 ± 13.45	162.31 ± 23.17	83	0.695	42	1.0E-02	139	1.4E-02	72	1.7E-01	2.5E-01	9.9E-03	5.0E-04
Amino Acid	Aspartate	116.76 ± 38.47	150.25 ± 50.87	114.23 ± 8.63	44.56 ± 8.74	38	0.032	30	2.5E-02	39	3.8E-06	35	2.4E-06	1.3E-03	4.7E-02	1.0E-04
Amino Acid	Glutamate	645.24 ± 158.32	912.89 ± 306.43	1399.97 ± 192.96	236.61 ± 43.72	37	0.012	26	2.2E-02	17	1.9E-04	23	1.0E-05	1.0E-04	2.0E-04	1.0E-04
Amino Acid	Glycine	280.93 ± 18.67	208.34 ± 50.79	275.1 ± 141.02	206.31 ± 54.61	73	0.053	99	9.6E-01	75	4.1E-01	80	2.3E-01	1.0E+00	1.4E-01	1.2E-01
Amino Acid	Hydroxyproline	-	-	-	248 ± 113	-	-	-	-	-	-	-	-	-	-	-
Amino Acid	Isoleucine	43.93 ± 17.08	58.12 ± 20.01	15.98 ± 1.67	17.53 ± 11.37	40	0.049	30	1.9E-02	110	8.0E-01	47	3.7E-02	9.4E-02	5.1E-01	3.8E-02
Amino Acid	Leucine	86.65 ± 28.27	76.32 ± 19.43	28.61 ± 4.26	49.26 ± 18.56	57	0.079	65	9.1E-02	172	9.4E-02	81	3.8E-01	5.9E-01	2.1E-02	4.5E-03
Amino Acid	Lysine	95.27 ± 64.51	195.73 ± 76.07	64.71 ± 3.92	63.92 ± 32.2	67	0.437	33	3.4E-02	99	9.6E-01	56	8.1E-02	5.0E-01	9.8E-01	5.5E-01
Amino Acid	Phenylalanine	51.55 ± 10.06	56.15 ± 18.15	17.56 ± 1.45	32.59 ± 5.68	63	0.025	58	7.9E-02	186	5.3E-03	82	2.8E-01	6.6E-01	4.1E-01	1.0E-04
Amino Acid	Phosphoserine	-	-	-	136 ± 61	-	-	-	-	-	-	-	-	-	-	-
Amino Acid	Tryptophan	12.32 ± 8.96	16.82 ± 3.67	3.69 ± 1.94	16.13 ± 3.1	131	0.479	96	7.9E-01	437	3.4E-04	155	4.8E-02	1.0E-04	4.0E-04	2.4E-01
Amino Acid	Tyrosine	28.8 ± 12.26	37.04 ± 8.87	13.44 ± 1.37	14.17 ± 7.72	49	0.102	38	7.6E-03	105	8.6E-01	56	5.9E-02	2.7E-01	7.4E-01	1.6E-01
Amino Acid	Valine	80.69 ± 31.52	89.44 ± 30.57	33.19 ± 1.56	33.08 ± 14.91	41	0.051	37	2.7E-02	100	9.9E-01	51	2.0E-02	5.8E-03	9.3E-01	6.1E-03
Organic Acid	Acetate	103.41 ± 17.93	71.85 ± 21.56	153.27 ± 77.42	16.41 ± 6.41	16	0.001	23	1.1E-02	11	2.5E-02	15	1.4E-04	4.1E-03	1.6E-01	8.0E-03
Organic Acid	Lactate	679.71 ± 163.38	637.37 ± 206.63	398.61 ± 109.54	322.78 ± 113.52	47	0.014	51	5.0E-02	81	3.7E-01	58	1.3E-02	1.8E-03	9.5E-02	4.8E-02
Organic Acid	Malate	109.37 ± 27.52	100.91 ± 3.39	115.46 ± 34.15	50.61 ± 18.48	46	0.015	50	5.3E-03	44	1.6E-02	46	7.9E-04	2.9E-01	7.5E-01	4.0E-04
Organic Acid	Nicotinate	10.91 ± 7.52	10.38 ± 3.32	9.52 ± 1.39	6.72 ± 4.01	62	0.382	65	2.1E-01	71	2.5E-01	66	1.9E-01	1.3E-01	4.3E-01	6.3E-01
Organic Acid	Propanoate	98.21 ± 35.27	84.34 ± 23.29	3.44 ± 1.77	3.27 ± 1.23	3	0.013	4	6.4E-03	95	8.8E-01	6	2.0E-03	1.4E-01	2.0E-04	1.3E-01
Organic Acid	Succinate	331.56 ± 44.15	287.17 ± 96.88	79.41 ± 14.69	26.94 ± 32.58	8	0.000	9	9.3E-03	34	3.1E-02	12	1.8E-04	5.3E-03	4.8E-03	9.5E-01
Nucleotide	NAD+	10.57 ± 4.76	18.98 ± 8.89	14.56 ± 2.82	2.3 ± 1.19	22	0.038	12	3.4E-02	16	3.8E-04	16	1.1E-05	2.0E-04	3.9E-02	2.9E-03
Carbohydrate	Glucose	124.21 ± 19.51	142.29 ± 55.48	98.04 ± 39.18	154.83 ± 14.75	125	0.050	109	6.9E-01	158	4.4E-02	129	2.6E-02	4.8E-02	6.5E-01	8.2E-01
Carbohydrate	Trehalose	185.95 ± 177.53	830.44 ± 233.98	229.94 ± 11.35	726.44 ± 241.7	391	0.010	87	5.6E-01	316	1.6E-02	181	6.5E-02	5.7E-03	4.8E-01	5.0E-03
Phospholipid-related	Betaine	253.1 ± 61.37	270.68 ± 102.41	206.77 ± 22.67	197.46 ± 59.07	78	0.243	73	2.8E-01	95	7.8E-01	82	2.6E-01	2.2E-02	3.3E-01	8.8E-03
Phospholipid-related	Choline	29.96 ± 19.82	18.73 ± 6.64	12.18 ± 2.11	64.6 ± 23.52	216	0.063	345	1.6E-02	530	1.2E-02	328	1.7E-02	1.0E-04	6.1E-01	8.4E-01
Phospholipid-related	GPC	49.76 ± 37.02	106.74 ± 19.63	64.61 ± 2.01	147.42 ± 25.64	296	0.006	138	4.3E-02	228	3.1E-03	202	1.5E-03	1.0E-04	1.6E-01	3.2E-01
Phospholipid-related	Glycerol	49.77 ± 17.85	294.51 ± 153.53	71.96 ± 10.29	19.17 ± 16.61	39	0.045	7	3.8E-02	27	1.3E-03	14	1.1E-02	1.0E-04	1.5E-02	3.1E-02
Phospholipid-related	O-Phosphocholine	43.76 ± 42.39	98.67 ± 25.97	100.09 ± 10.6	229.29 ± 43.83	524	0.001	232	2.1E-03	229	3.5E-03	279	1.1E-03	1.0E-04	1.0E-04	1.0E-04

SI Table 1, part 4

Experiment 2
Comparison N2 v. long-lived mutants, old adults (240h), 25.0oC
Data Relative concentrations determined from NMR spectra and computer-assisted manual fitting of metabolites
Analysis (1) a t-test of each mutant v. N2; (2) Fisher combined probability tests (FCP) of IIS mutants, with and without daf-28

	**Genotype**	N2	*daf-2(e1370)*			*daf-2(m41)*			*daf-2(m596)*			*daf-28(sa191)*			daf-2(-) only	all IIS	ife-2(ok306)		
	**Statistic**	Mean ± 95%CI	Mean ± 95%CI	% change	P, t-test	Mean ± 95%CI	% change	P, t-test	Mean ± 95%CI	% change	P, t-test	Mean ± 95%CI	% change	P, t-test	FCP	FCP	Mean ± 95%CI	% change	P, t-test
**Compound Class**	**Compound**																		
Amino Acid	Alanine	879.64 ± 32.59	922.1 ± 37.84	105	1.4E-01	1114.56 ± 218.85	127	7.1E-02	758.76 ± 87.53	86	2.2E-02	806.11 ± 74.15	92	1.1E-01	7.3E-02	2.9E-02	1026.92 ± 74.48	117	1.4E-02
Amino Acid	Arginine	116.37 ± 13.45	126.24 ± 29.45	108	5.4E-01	75.52 ± 25.17	65	2.3E-02	152.07 ± 25.62	131	3.6E-02	195.95 ± 21.36	168	2.7E-04	9.3E-08	1.1E-06	155.44 ± 6.08	134	1.0E-04
Amino Acid	Aspartate	114.23 ± 8.63	80.94 ± 7.24	71	8.3E-04	127.15 ± 18.29	111	2.5E-01	28.34 ± 5.02	25	8.5E-06	74.53 ± 12.78	65	1.0E-03	3.3E-12	4.5E-08	170.87 ± 16.96	150	5.0E-04
Amino Acid	Glutamate	1399.97 ± 192.96	1454.93 ± 56.09	104	6.5E-01	450.03 ± 61.29	32	1.6E-05	355.19 ± 43.62	25	2.2E-04	337.72 ± 28.69	24	5.2E-06	2.8E-04	1.8E-02	372.64 ± 33.44	27	4.9E-08
Amino Acid	Glycine	275.1 ± 141.02	139.25 ± 15.6	51	1.4E-01	172.84 ± 18.11	63	2.0E-01	186.16 ± 28.78	68	3.9E-01	114.99 ± 33.03	42	6.2E-02	4.7E-08	1.9E-06	128.87 ± 9.26	47	2.3E-02
Amino Acid	Hydroxyproline	-	-	-	-	-	-	-	-	-	-	-	-	-	-	-	-	-	-
Amino Acid	Isoleucine	15.98 ± 1.67	26.32 ± 4.34	165	2.1E-03	88.98 ± 13.31	557	5.2E-06	32.41 ± 6.93	203	1.1E-03	50.31 ± 7.41	315	2.1E-05	3.2E-15	7.4E-16	29.42 ± 3.6	184	1.9E-04
Amino Acid	Leucine	28.61 ± 4.26	50.74 ± 10.83	177	4.8E-03	137.1 ± 15.01	479	8.1E-07	61.4 ± 12.85	215	1.1E-03	94.03 ± 15.26	329	4.0E-05	3.8E-17	5.9E-17	47.63 ± 3.16	166	1.9E-05
Amino Acid	Lysine	64.71 ± 3.92	99.94 ± 10.02	154	2.1E-04	108.52 ± 22.22	168	5.2E-03	86.46 ± 11.46	134	5.0E-03	112.62 ± 6.83	174	2.3E-06	3.3E-13	6.8E-14	98.3 ± 10.42	152	5.6E-04
Amino Acid	Phenylalanine	17.56 ± 1.45	28.59 ± 4.56	163	1.6E-03	65.2 ± 5.33	371	1.5E-07	35.6 ± 1.51	203	4.0E-06	43.73 ± 3.58	249	9.9E-07	1.4E-24	2.6E-23	35.29 ± 2.46	201	5.2E-07
Amino Acid	Phosphoserine	-	-	-	-	-	-	-	-	-	-	-	-	-	-	-	-	-	-
Amino Acid	Tryptophan	3.69 ± 1.94	5.75 ± 1.14	156	1.4E-01	17.12 ± 0.94	464	1.9E-06	8.52 ± 4.58	231	6.7E-02	14.46 ± 1.25	392	1.6E-05	1.2E-18	3.1E-16	14.7 ± 0.65	399	6.9E-08
Amino Acid	Tyrosine	13.44 ± 1.37	20.15 ± 2.57	150	2.0E-03	47.4 ± 3.71	353	1.6E-07	22.36 ± 1.93	166	2.7E-04	29.89 ± 3.9	222	5.3E-05	1.0E-18	1.9E-19	26.18 ± 1.9	195	1.4E-06
Amino Acid	Valine	13.44 ± 1.38	52.34 ± 8.28	158	1.5E-03	126.82 ± 12.33	382	4.3E-07	58.9 ± 3.3	177	4.1E-06	75.09 ± 8.83	226	1.6E-05	1.9E-18	3.0E-19	50.94 ± 4.99	153	2.4E-04
Organic Acid	Acetate	153.27 ± 77.42	37.28 ± 4.69	24	3.6E-02	92.37 ± 14.24	60	1.7E-01	20.08 ± 5.98	13	4.5E-02	18.63 ± 3.77	12	9.3E-03	1.4E-06	1.0E-05	48.75 ± 7.79	32	6.0E-03
Organic Acid	Lactate	398.61 ± 109.54	297.66 ± 51.64	75	1.8E-01	438.24 ± 59.73	110	5.5E-01	291.24 ± 34.12	73	2.1E-01	281.09 ± 47.47	71	9.0E-02	5.6E-02	6.6E-02	346.72 ± 52.17	87	3.7E-01
Organic Acid	Malate	115.46 ± 34.15	58.44 ± 21.23	51	3.5E-02	115.49 ± 28.52	100	1.0E+00	57.75 ± 5	50	4.8E-02	72.65 ± 14.08	63	5.3E-02	5.5E-05	5.7E-04	58.17 ± 26.96	50	2.6E-02
Organic Acid	Nicotinate	9.52 ± 1.39	12.39 ± 2.11	130	5.4E-02	29.91 ± 5.91	314	1.7E-04	9.43 ± 2.07	99	9.5E-01	17.23 ± 1.62	181	1.0E-04	1.5E-08	2.6E-09	27.38 ± 6	288	9.1E-04
Organic Acid	Propanoate	3.44 ± 1.77	8.84 ± 2.17	257	6.6E-03	22.64 ± 6.88	658	7.3E-04	4.86 ± 2.05	141	3.6E-01	10.41 ± 3.75	303	1.1E-02	2.0E-08	5.2E-09	68.25 ± 16.83	1984	1.1E-04
Organic Acid	Succinate	79.41 ± 14.69	160.37 ± 18.03	202	2.3E-04	300.63 ± 54.33	379	5.7E-05	120.76 ± 39.69	152	6.0E-02	85.75 ± 15.44	108	5.8E-01	1.9E-09	2.8E-10	202.66 ± 32.43	255	1.6E-04
Nucleotide	NAD+	14.56 ± 2.82	12.4 ± 2.84	85	3.3E-01	2.37 ± 0.55	16	3.3E-05	1.19 ± 1.23	8	5.0E-04	3.43 ± 1.8	24	1.8E-04	2.2E-15	5.5E-13	6.48 ± 1.07	44	7.0E-05
Carbohydrate	Glucose	98.04 ± 39.18	112.43 ± 8.99	115	5.5E-01	384.55 ± 77.8	392	2.0E-04	145.14 ± 18.53	148	1.4E-01	128.9 ± 9.09	131	1.7E-01	5.3E-20	2.4E-17	109.37 ± 15.81	112	5.5E-01
Carbohydrate	Trehalose	229.94 ± 11.35	318.22 ± 76.3	138	3.9E-02	268.01 ± 47.6	117	1.7E-01	603.75 ± 107.23	263	9.4E-05	521.66 ± 162.13	227	7.9E-03	7.9E-12	7.4E-11	629.99 ± 71.03	274	3.5E-06
Phospholipid-related	Betaine	206.77 ± 22.67	306.33 ± 94.65	148	6.0E-02	226.42 ± 40.93	110	4.3E-01	99.5 ± 5.63	48	4.6E-04	101.76 ± 28.87	49	5.1E-04	5.7E-07	2.2E-07	142.38 ± 12.27	69	2.3E-04
Phospholipid-related	Choline	12.18 ± 2.11	27.76 ± 6.59	228	1.8E-03	184.59 ± 33.92	1515	8.9E-06	53.72 ± 7.35	441	1.1E-05	73.22 ± 10.83	601	4.6E-06	1.5E-19	1.4E-18	50.03 ± 9.32	411	7.4E-05
Phospholipid-related	GPC	64.61 ± 2.01	53.53 ± 5.2	83	3.8E-03	120.44 ± 18.21	186	3.3E-04	64.52 ± 7.52	100	9.8E-01	85.73 ± 25.83	133	1.5E-01	8.2E-05	3.9E-04	174.48 ± 23.57	270	2.0E-05
Phospholipid-related	Glycerol	71.96 ± 10.29	29.16 ± 2.3	41	2.0E-04	114.34 ± 22.13	159	9.3E-03	84.17 ± 11.44	117	1.9E-01	60.1 ± 13.05	84	2.0E-01	2.6E-03	2.5E-03	80.12 ± 8.9	111	2.7E-01
Phospholipid-related	O-Phosphocholine	100.09 ± 10.6	56.32 ± 9.15	56	5.8E-04	96.57 ± 30.8	96	8.4E-01	32.93 ± 4.36	33	1.0E-04	74.55 ± 37.39	74	2.3E-01	6.2E-05	1.4E-05	128.24 ± 18.27	128	4.9E-02

SI Table 1, part 5

Experiment 3
Comparison N2 v. daf-2(e1380), or daf-16(m26) or daf-2(e1370);daf-16(m26) at 22.5oC
Data Relative concentrations determined from NMR spectra and computer-assisted manual fitting of metabolites
Analysis t-test

	**Genotype**	N2	*daf-2(e1370)*			*daf-16(m26)*			*daf-2(e1370);daf-16(m26)*		
	**Statistic**	Mean ± 95%CI	Mean ± 95%CI	% change	P, t-test	Mean ± 95%CI	% change	t-test	Mean ± 95%CI	% change	P, t-test
**Compound Class**	**Compound**										
Amino Acid	Alanine	117.85 ± 7.99	126.52 ± 18.47	107	3.8E-01	87.6 ± 5.63	74	9.9E-06	100.84 ± 11.57	86	2.7E-02
Amino Acid	Arginine	22.57 ± 2.85	21.68 ± 3.57	96	7.0E-01	20.61 ± 3.61	91	4.1E-01	24.32 ± 2.36	108	3.7E-01
Amino Acid	Aspartate	9.97 ± 0.73	9.39 ± 2.33	94	6.1E-01	11.49 ± 0.99	115	2.6E-02	11.74 ± 1.28	118	2.7E-02
Amino Acid	Glutamate	133.58 ± 10.79	138.8 ± 33.69	104	7.6E-01	138.01 ± 10.07	103	5.6E-01	178.38 ± 15.28	134	1.8E-04
Amino Acid	Glycine	17.35 ± 2.22	13.12 ± 2.26	76	2.0E-02	14.83 ± 1.95	85	1.1E-01	14.63 ± 0.86	84	4.7E-02
Amino Acid	Hydroxyproline	-	-	-	-	-	-	-	-	-	-
Amino Acid	Isoleucine	3.28 ± 0.34	5.67 ± 0.8	173	3.0E-05	3.93 ± 0.59	120	8.0E-02	2.62 ± 0.67	80	9.3E-02
Amino Acid	Leucine	6.22 ± 0.48	11.28 ± 3.04	181	2.3E-03	7.21 ± 0.41	116	6.5E-03	6.39 ± 1.05	103	7.7E-01
Amino Acid	Lysine	3.62 ± 1.25	6.6 ± 1.14	183	3.9E-03	9.35 ± 2.36	258	5.3E-04	3.87 ± 1.25	107	7.9E-01
Amino Acid	Phenylalanine	6.63 ± 0.7	4.04 ± 0.68	61	1.0E-04	5.25 ± 0.38	79	3.2E-03	4.97 ± 0.47	75	1.5E-03
Amino Acid	Phosphoserine	-	-	-	-	-	-	-	-	-	-
Amino Acid	Tryptophan	1.27 ± 0.41	0.88 ± 0.46	70	2.4E-01	1.24 ± 0.24	98	9.1E-01	0.8 ± 0.3	64	1.0E-01
Amino Acid	Tyrosine	2.88 ± 0.14	2.59 ± 0.31	90	9.8E-02	2.31 ± 0.18	80	1.4E-04	2.44 ± 0.27	85	1.1E-02
Amino Acid	Valine	5.77 ± 0.41	12.29 ± 1.67	213	3.8E-07	5.96 ± 0.47	103	5.5E-01	6.67 ± 1.47	116	2.4E-01
Organic Acid	Acetate	9.24 ± 1.58	7.15 ± 0.92	77	5.3E-02	6.73 ± 0.62	73	9.7E-03	6.98 ± 0.64	76	2.4E-02
Organic Acid	Lactate	38.63 ± 6.43	21.73 ± 2.04	56	4.1E-04	26.29 ± 3.2	68	3.5E-03	22.72 ± 2.8	59	5.2E-04
Organic Acid	Malate	8.33 ± 0.98	6.24 ± 1.77	75	4.8E-02	6.83 ± 0.303	82	1.0E-02	9.86 ± 1.045	118	5.2E-02
Organic Acid	Nicotinate	0.48 ± 0.13	0.37 ± 0.2	78	4.0E-01	0.42 ± 0.08	87	4.4E-01	0.38 ± 0.11	79	2.8E-01
Organic Acid	Propanoate	2.56 ± 0.95	1.67 ± 0.53	65	1.6E-01	1.6 ± 0.3	63	7.6E-02	1.15 ± 0.26	45	1.6E-02
Organic Acid	Succinate	26.24 ± 5.99	15.68 ± 4.86	60	2.0E-02	22.98 ± 2.77	88	3.5E-01	16.9 ± 1	64	1.1E-02
Nucleotide	NAD+	1.82 ± 0.24	1.22 ± 0.55	67	5.3E-02	1.7 ± 0.21	93	4.6E-01	1.1 ± 0.62	61	4.2E-02
Carbohydrate	Glucose	8.2 ± 1.33	7.47 ± 1.52	91	4.9E-01	7.93 ± 1.28	97	7.8E-01	8.73 ± 2.22	106	6.9E-01
Carbohydrate	Trehalose	33.45 ± 4.96	58.64 ± 14.08	175	2.4E-03	37.29 ± 3.21	111	2.2E-01	62.23 ± 9.26	186	3.7E-05
Phospholipid-related	Betaine	24.85 ± 1.13	27.45 ± 4.9	110	2.8E-01	28.35 ± 1.98	114	7.6E-03	38.28 ± 3.08	154	2.0E-07
Phospholipid-related	Choline	1.59 ± 0.5	2.14 ± 0.53	134	1.7E-01	2.79 ± 0.92	175	3.9E-02	2.29 ± 1.38	144	3.4E-01
Phospholipid-related	GPC	1.36 ± 0.81	1.73 ± 0.72	127	5.3E-01	2.13 ± 0.52	157	1.3E-01	1.57 ± 0.7	115	7.1E-01
Phospholipid-related	Glycerol	6.71 ± 0.69	5.23 ± 0.67	78	9.2E-03	4.92 ± 1.14	73	1.7E-02	5.6 ± 0.92	83	7.2E-02
Phospholipid-related	O-Phosphocholine	4.53 ± 0.66	2.13 ± 0.25	47	1.7E-05	4.03 ± 1.18	89	4.8E-01	5.05 ± 0.96	112	3.8E-01
