# Supplementary material for: A metabolic signature of long life in Caenorhabditis elegans
Source: BMC Biol. 2010 Feb 10;8:14. doi: 10.1186/1741-7007-8-14 (PMC2829508; doi:10.1186/1741-7007-8-14)
Supplement: Additional file 2 — Table S2. Expression of genes encoding enzymes in selected metabolic pathways. Relative expression in daf-2(e1370) adults compared to wild-type worms; data from reference [20]. [file 1741-7007-8-14-S2.PDF]

pathway	enzyme number	enzyme name	Sequence name	Gene Public Name	WBGene	Exp 9	Exp 7	Exp 8	Exp 1	Exp 2	Exp 3	Exp4	Exp 5	Exp 6	mean	stdev	count	t-statistic	pvalue
citric acid cycle	1.1.1.42	isocitrate dehydrogenoase	C34F6.8		WBGene00007942	0.08	0.23	-0.14	-0.09	0.19	0.03	0.26	-0.01	-0.07	0.05	0.15	9	1.09	0.3056
citric acid cycle	1.1.1.42	isocitrate dehydrogenoase	F59B8.2		WBGene00010317	0.02	-0.20	-0.34	0.59	0.27	0.19	0.08	-0.29	-0.31	0.00	0.32	9	0.01	0.9918
citric acid cycle	1.1.1.42	isocitrate dehydrogenoase	C37E2.1		WBGene00007993	0.21	-0.06	0.47	0.69	0.52	0.38	0.44	0.34	0.18	0.35	0.22	9	4.82	0.0013
citric acid cycle	1.1.1.42	isocitrate dehydrogenoase	C30F12.7		WBGene00016266	1.14	0.66	0.64	1.96	1.45	1.14	1.32	1.06	0.97	1.15	0.41	9	8.50	0.0000
citric acid cycle	1.2.4.2	alpha-ketoglutrate dehydrogenoase	T22B11.5		WBGene00020679	-0.35	0.54	-0.31	-0.54	0.23	0.27	-0.05	-0.18	-0.21	-0.07	0.35	9	-0.58	0.5809
citric acid cycle	1.2.4.3	alpha-ketoglutrate dehydrogenoase	ZK836.2		WBGene00014098	-0.14	-0.11	-0.33	-0.77	-0.19	-0.12	-0.28	-0.23	-0.13	-0.26	0.21	9	-3.69	0.0061
citric acid cycle	1.2.4.4	alpha-ketoglutrate dehydrogenoase	WO2F12.5		WBGene00020950	0.19	-0.26	0.13	0.30	-0.31	-0.14	-0.15	-0.18	0.00	-0.05	0.21	9	-0.66	0.5287
citric acid cycle	1.2.4.5	alpha-ketoglutrate dehydrogenoase	LLC1.3		WBGene00010794	-0.10	-0.26	-0.16	-0.08	-0.04	0.13	-0.28	-0.20	-0.21	-0.13	0.13	9	-3.13	0.0140
citric acid cycle	1.3.5.1	succino datate dehydrogenoase	C03G5.1	sdha-1	WBGene00015391	-0.15	0.04	0.02	0.12	0.61	0.29	0.19	0.06	-0.10	0.12	0.23	9	1.58	0.1527
citric acid cycle	1.3.5.1	succino datate dehydrogenoase	C34B2.7	sdha-2	WBGene00016392	-0.18	-0.13	-0.31	-0.69	-0.55	-0.23	-0.25	-0.57	-0.40	-0.37	0.20	9	-5.63	0.0005
citric acid cycle	1.3.5.1	succino datate dehydrogenoase	F33A8.5	sdhd-1	WBGene00009353	-0.23	0.18	-0.23	0.05	0.07	0.27	0.09	-0.20	-0.27	-0.03	0.20	9	-0.44	0.6701
citric acid cycle	1.3.5.1	succino datate dehydrogenoase	F42A8.2	sdhb-1	WBGene00006433	0.04	0.31	-0.05	-0.06	0.31	0.14	0.20	-0.12	-0.08	0.08	0.17	9	1.36	0.2096
citric acid cycle	1.3.5.1	succino datate dehydrogenoase	T07C4.7	mev-1	WBGene00003225	-0.16	0.16	-0.20	0.10	0.35	0.16	0.08	-0.14	-0.16	0.02	0.19	9	0.33	0.7508
citric acid cycle	2.3.3.1	citrate synthase	T20G5.2	cts-1	WBGene00000833	0.08	-0.15	-0.10	0.60	0.10	-0.04	-0.11	-0.30	-0.17	-0.01	0.26	9	-0.12	0.9110
citric acid cycle	4.2.1.2	fumarase	H14A12.2	fum-1	WBGene00001503	-0.17	0.47	-0.29	-0.74	0.33	0.12	-0.11	-0.17	-0.21	-0.09	0.36	9	-0.72	0.4935
citric acid cycle	4.2.1.3	acontinoase	F43C1.6		WBGene00023422	no data	no data	no data	no data	no data	no data	no data	no data	no data	-	-	0	-	-
citric acid cycle	4.2.1.3	acontinoase	F54H12.1	aco-2	WBGene00000041	-0.13	0.19	0.18	-0.44	-0.01	-0.23	-0.16	-0.47	-0.27	-0.15	0.24	9	-1.88	0.0970
citric acid cycle	4.2.1.3	acontinoase	ZK455.1	aco-1	WBGene00000040	-0.12	-0.43	-0.77	0.03	0.14	0.13	-0.27	-0.22	-0.39	-0.21	0.30	9	-2.14	0.0650
citric acid cycle	6.2.1.5	succinyl-CoA synthetase	F47B10.1		WBGene00009812	0.13	0.02	-0.09	0.14	0.05	0.09	-0.14	-0.18	-0.12	-0.01	0.12	9	-0.27	0.7931
citric acid cycle/glyoxylate shunt	1.1.1.37	cytosolic malate dehydrogenoase	F20H11.3	mdh-1	WBGene00003162	-0.26	-0.15	-0.34	0.15	-0.08	0.16	-0.08	-0.38	-0.41	-0.15	0.21	9	-2.17	0.0617
citric acid cycle/glyoxylate shunt	1.1.1.37	cytosolic malate dehydrogenoase	F36A2.3		WBGene00009453	0.58	0.09	-0.28	0.81	0.42	0.45	0.56	0.13	0.14	0.32	0.33	9	2.93	0.0191
citric acid cycle/glyoxylate shunt	1.1.1.37	cytosolic malate dehydrogenoase	F46E10.10		WBGene00018491	0.27	0.16	0.35	0.78	0.23	0.17	-0.05	0.27	0.21	0.27	0.22	9	3.58	0.0072
citric acid cycle/glyoxylate shunt	1.1.1.37	cytosolic malate dehydrogenoase	T01B6.3		WBGene00020135	1.77	1.65	1.71	2.48	1.91	1.62	1.69	1.53	1.59	1.77	0.29	9	18.50	0.0000
citric acid cycle/glyoxylate shunt	1.1.1.37	cytosolic malate dehydrogenoase	VF13D12L.3		WBGene00012149	0.14	-0.15	-0.21	0.72	0.85	0.47	0.11	0.03	-0.02	0.22	0.38	9	1.71	0.1255
glyoxylate shunt	1.2.1.3	aldehyde dehydrogenoase	F01F1.6	alh-9	WBGene00000115	0.80	0.39	0.67	1.39	0.70	0.95	0.90	0.82	0.67	0.81	0.27	9	8.93	0.0000
glyoxylate shunt	1.2.1.3	aldehyde dehydrogenoase	F54D8.3	alh-1	WBGene00000107	0.41	0.11	0.17	0.99	0.37	0.30	0.10	0.11	0.06	0.29	0.29	9	3.00	0.0171
glyoxylate shunt	1.2.1.5	aldehyde dehydrogenoase	T08B1.3	alh-5	WBGene00000111	1.11	0.22	0.30	0.28	1.18	0.73	1.28	0.93	0.99	0.78	0.42	9	5.63	0.0005
glyoxylate shunt	4.1.3.1	malate synthase /isocitrate lyase	C05E4.9	gei-7	WBGene00001564	1.70	1.48	1.64	1.89	2.69	0.04	1.30	0.92	0.85	1.39	0.75	9	5.59	0.0005
glyoxylate shunt	6.2.1.1	acetyl-CoA synthetase	C36A4.9		WBGene00007969	0.49	0.03	0.35	2.58	-0.10	-0.15	-0.24	0.10	0.04	0.34	0.87	9	1.19	0.2690
glyoxylate shunt	6.2.1.1	acetyl-CoA synthetase	K03A1.5	sur-5	WBGene00006351	0.08	-0.38	-0.39	0.39	0.23	0.43	0.08	-0.01	-0.25	0.02	0.31	9	0.19	0.8503
glyoxylate shunt	6.2.1.1	acetyl-CoA synthetase	ZK112.6		WBGene00022662	no data	no data	no data	no data	no data	no data	no data	no data	no data	-	-	0	-	-
gluconeogenesis	2.7.9.2	phosphoenolpyruvate synthase	T21C9.6		WBGene00011893	0.18	-0.07	-0.07	0.44	0.13	0.44	0.50	0.30	-0.19	0.18	0.25	9	2.18	0.0610
gluconeogenesis	3.1.3.11	fructose-bisphosphatase	K07A3.1	fbp-1	WBGene00001404	0.24	0.22	0.14	0.62	0.59	0.50	0.26	0.31	0.15	0.34	0.19	9	5.46	0.0006
gluconeogenesis	4.1.1.32	phosphoenolpyruvate carboxykinoase	H04M03.1		WBGene00019151	0.03	-0.06	-0.22	-0.56	0.65	0.42	-0.12	-0.03	-0.06	0.01	0.35	9	0.05	0.9633
gluconeogenesis	4.1.1.32	phosphoenolpyruvate carboxykinoase	R11A5.4		WBGene00011232	1.50	0.38	1.09	2.36	1.66	0.85	1.27	1.51	1.49	1.35	0.55	9	7.30	0.0001
gluconeogenesis	4.1.1.32	phosphoenolpyruvate carboxykinoase	W05G11.6		WBGene00021043	no data	-0.11	0.13	1.50	0.55	no data	0.28	-0.22	-0.42	0.24	0.64	7	1.01	0.3524
gluconeogenesis	6.4.1.1	pyruvate carboxylase	D2023.2	pyc-1	WBGene00004258	0.19	0.14	0.19	0.88	0.38	0.09	0.18	0.00	0.04	0.23	0.27	9	2.62	0.0308
glycolysis/neoglucogenesis	1.2.1.12	glyceraldehyde-3-phosphate dehydrogenoase	F33H1.2	gpd-4	WBGene00001686	no data	no data	no data	no data	no data	no data	no data	no data	no data	-	-	0	-	-
glycolysis/neoglucogenesis	1.2.1.12	glyceraldehyde-3-phosphate dehydrogenoase	K10B3.7	gpd-3	WBGene00001685	1.23	0.70	0.80	1.53	1.17	0.79	1.08	0.99	0.94	1.03	0.26	9	11.83	0.0000
glycolysis/neoglucogenesis	1.2.1.12	glyceraldehyde-3-phosphate dehydrogenoase	T09F3.3	gpd-1	WBGene00001683	0.00	0.01	0.06	-0.41	-0.49	-0.31	-0.38	-0.17	-0.01	-0.19	0.21	9	-2.67	0.0284
glycolysis/neoglucogenesis	1.2.1.12	glyceraldehyde-3-phosphate dehydrogenoase	K10B3.8	gpd-2	WBGene00001684	no data	no data	no data	no data	no data	no data	no data	no data	no data	-	-	-	-	-
glycolysis/neoglucogenesis	2.7.2.3	phosphoglycerate kinoase	T03F1.3	pgk-1	WBGene00020185	0.25	0.06	0.23	0.87	0.02	0.10	-0.07	0.10	0.11	0.19	0.27	9	2.03	0.0772
glycolysis/neoglucogenesis	4.1.2.13	fructose-bisphosphate aldolase	F01F1.12		WBGene00017166	0.47	-0.11	0.42	1.24	-0.06	-0.08	-0.15	0.30	0.44	0.27	0.45	9	1.85	0.1015
glycolysis/neoglucogenesis	4.1.2.13	fructose-bisphosphate aldolase	T05D4.1		WBGene00011474	0.40	0.15	0.20	0.83	0.68	0.44	0.43	0.18	0.29	0.40	0.23	9	5.18	0.0008
glycolysis/neoglucogenesis	4.2.1.11	enolase	T21B10.2	enol-1	WBGene00011884	0.46	-0.08	0.50	1.02	0.01	-0.02	-0.11	0.32	0.32	0.27	0.37	9	2.20	0.0590
glycolysis/neoglucogenesis	5.3.1.9	glucose-6-phosphate isomerase	Y87G2A.8	gpi-1	WBGene00013597	0.04	0.13	0.01	-1.00	-0.18	-0.02	-0.17	-0.09	-0.08	-0.15	0.33	9	-1.36	0.2110
glycolysis/neoglucogenesis	5.4.2.1	phosphoglycerate mutase	F57B10.3		WBGene00019001	0.21	0.27	0.18	0.77	0.06	0.30	0.22	no data	-0.01	0.25	0.23	8	3.02	0.0195
glycolysis/neoglucogenesis	5.4.2.1	phosphoglycerate mutase	R07G3.5		WBGene00019941	-0.11	0.04	-0.18	-0.41	-0.50	-0.25	-0.28	-0.42	-0.30	-0.27	0.17	9	-4.78	0.0014
glycolysis/neoglucogenesis	5.4.2.1	phosphoglycerate mutase	T07F12.1		WBGene00020321	0.08	-0.32	-0.17	0.09	0.01	-0.13	0.26	0.05	0.30	0.02	0.20	9	0.28	0.7840
glycolysis/neoglucogenesis	5.4.2.1	phosphoglycerate mutase	Y18H1A.4		WBGene00021210	0.04	-0.17	-0.13	-0.52	0.14	0.03	-0.14	-0.19	-0.14	-0.12	0.19	9	-1.91	0.0924
glycolysis/neoglucogenesis	5.4.2.1	phosphoglycerate mutase	ZK484.6		WBGene00022752	no data	-0.49	0.07	0.01	-0.01	0.30	-0.58	no data	0.26	-0.06	0.34	7	-0.48	0.6463
glycolysis/neoglucogenesis	5.4.2.2	phosphoglucomutase	R05F9.6		WBGene00019890	-0.04	0.06	-0.23	0.70	-0.43	0.00	-0.07	-0.26	-0.04	-0.03	0.32	9	-0.33	0.7517
glycolysis	1.2.4.1	pyruvate dehydrogenoase	C04C3.3		WBGene00015413	-0.25	-0.06	-0.29	-0.29	-0.25	-0.03	-0.22	-0.27	-0.29	-0.22	0.10	9	-6.48	0.0002
glycolysis	1.2.4.1	pyruvate dehydrogenoase	F10G7.1	tag-151	WBGene00006497	-0.18	0.25	-0.30	-0.96	-0.21	0.03	-0.18	-0.41	-0.34	-0.26	0.33	9	-2.31	0.0495
glycolysis	1.2.4.1	pyruvate dehydrogenoase	T05H10.6		WBGene00011510	-0.18	-0.07	-0.41	-0.42	-0.28	-0.08	-0.10	-0.33	-0.29	-0.24	0.14	9	-5.24	0.0008
glycolysis	1.2.4.1	pyruvate dehydrogenoase	Y61A9LA.10		WBGene00022021	-0.21	-0.07	-0.11	-0.83	-0.03	0.12	-0.22	-0.29	-0.30	-0.22	0.27	9	-2.42	0.0416
glycolysis	1.8.1.4	pyruvate dehydrogenoase	LLC1.3		WBGene00010794	-0.10	-0.26	-0.16	-0.08	-0.04	0.13	-0.28	-0.20	-0.21	-0.13	0.13	9	-3.13	0.0140
glycolysis	2.3.1.12	pyruvate dehydrogenoase	C30H6.7		WBGene00007824	-0.04	-0.06	-0.22	-0.32	-0.10	-0.03	-0.15	-0.34	-0.21	-0.16	0.12	9	-4.20	0.0030
glycolysis	2.3.1.12	pyruvate dehydrogenoase	F23B12.5		WBGene00009082	-0.08	0.25	-0.27	-0.47	-0.21	-0.09	-0.25	-0.19	-0.11	-0.16	0.19	9	-2.44	0.0407
glycolysis	2.3.1.12	pyruvate dehydrogenoase	ZK669.4		WBGene00014054	-0.27	-0.29	-0.41	-0.61	-0.25	-0.10	-0.44	-0.38	-0.47	-0.36	0.15	9	-7.23	0.0001
glycolysis	2.7.1.1	hexokinoase	F14B4.2		WBGene00008780	-0.68	0.57	-0.45	-1.05	-0.34	-0.18	-0.24	-0.67	-0.51	-0.39	0.45	9	-2.64	0.0297
glycolysis	2.7.1.1	hexokinoase	H25P06.1		WBGene00010416	-0.14	0.57	-0.12	-0.30	0.08	-0.14	-0.10	-0.22	0.03	-0.04	0.26	9	-0.44	0.6688
glycolysis	2.7.1.1	hexokinoase	Y77E11A.1		WBGene00022306	-0.23	0.26	-0.19	-0.33	-0.07	-0.23	0.01	-0.33	-0.25	-0.15	0.19	9	-2.38	0.0443
glycolysis	2.7.1.11	6-phosphofructokinoase	C50F4.2		WBGene00008230	-0.06	-0.54	-0.10	0.13	0.68	0.97	-0.36	0.16	0.15	0.11	0.47	9	0.73	0.4881
glycolysis	2.7.1.11	6-phosphofructokinoase	Y71H10A.1		WBGene00022199	0.06	0.41	0.17	0.33	0.43	0.42	0.35	0.15	0.10	0.27	0.15	9	5.45	0.0006
glycolysis	2.7.1.40	pyruvate kinoase	F25H5.3		WBGene00009126	0.01	0.01	-0.09	0.30	-0.03	0.13	0.01	-0.34	-0.26	-0.03	0.19	9	-0.45	0.6621
glycolysis	2.7.1.40	pyruvate kinoase	F44E2.7		WBGene00018420	-0.43	0.34	-0.18	-0.59	-0.19	-0.05	-0.19	-0.32	-0.14	-0.19	0.26	9	-2.25	0.0545
glycolysis	2.7.1.40	pyruvate kinoase	ZK593.1		WBGene00014001	0.64	0.45	0.20	1.22	0.45	0.76	0.51	0.59	0.24	0.56	0.30	9	5.54	0.0005
Starch metabolism	2.4.1.11	glycogen(starch) synthase	Y46G5A.31	gsy-1	WBGene00001793	0.10	-0.23	0.08	0.41	-0.06	-0.01	0.16	-0.27	-0.03	0.02	0.21	9	0.24	0.8139
Starch metabolism	2.4.1.1	glycogen phosphorylase	T22F3.3		WBGene00020696	0.01	1.49	0.07	-0.89	0.30	0.37	0.09	-0.12	0.20	0.17	0.62	9	0.82	0.4356
Starch metabolism	3.1.3.12	trehalose-6-phosphatase	H13N06.3	gob-1	WBGene00001649	0.18	0.57	-0.04	0.91	0.66	0.33	0.30	0.16	0.22	0.37	0.29	9	3.73	0.0058

Starch metabolism	2.4.1.15	trehalose-phosphate synthase	F19H8.1	tps-2	WBGene00006603	0.75	0.70	0.36	1.57	0.62	0.76	0.82	0.54	0.05	0.69	0.41	9	5.01	0.0010
Starch metabolism	2.4.1.15	trehalose-phosphate synthase	ZK54.2	tps-1	WBGene00006602	0.85	0.60	0.89	1.83	0.59	0.41	1.11	0.85	0.89	0.89	0.41	9	6.53	0.0002
Starch metabolism	3.2.1.28	trehalase	C23H3.7	tre-5	WBGene00006611	0.04	-0.11	0.20	-1.55	-0.19	-0.35	-0.12	-0.10	0.07	-0.23	0.52	9	-1.36	0.2119
Starch metabolism	3.2.1.28	trehalase	F15A2.2	tre-4	WBGene00006610	1.02	0.67	0.66	1.76	0.98	0.72	0.99	0.87	0.58	0.92	0.36	9	7.73	0.0001
Starch metabolism	3.2.1.28	trehalase	F57B10.7	tre-1	WBGene00006607	-0.31	-0.08	-0.22	-0.16	-0.03	-0.03	-0.24	-0.67	-0.58	-0.26	0.23	9	-3.36	0.0099
Starch metabolism	3.2.1.28	trehalase	T05A12.2	tre-2	WBGene00006608	-0.22	-0.01	-0.40	0.11	-0.30	0.00	-0.15	-0.20	-0.33	-0.17	0.17	9	-2.94	0.0188
Starch metabolism	3.2.1.28	trehalase	W05E10.4	tre-3	WBGene00006609	-0.14	-0.23	-0.37	-0.64	0.08	-0.33	0.11	-0.01	-0.41	-0.22	0.25	9	-2.60	0.0318
anaerobic fermentation	1.1.1.40	malic enzyme	Y48B6A.12		WBGene00012983	-0.36	0.57	-0.36	-0.76	-0.01	0.10	-0.15	-0.33	-0.19	-0.17	0.37	9	-1.34	0.2158
anaerobic fermentation	1.3.1.6	fumarate reductase (soluble)	F48E8.3		WBGene00018610	0.16	-0.40	0.34	0.59	-0.32	-0.33	-0.24	0.13	0.13	0.01	0.34	9	0.06	0.9552
tRNA-Ala	6.1.1.13	Alanyl-tRNA synthetase	W02B12.6	ars-1	WBGene00000196	-0.09	0.10	0.04	-0.48	-0.43	-0.07	-0.02	-0.30	-0.16	-0.16	0.20	9	-2.30	0.0507
tRNA-Ala	6.1.1.13	Alanyl-tRNA synthetase	F28H1.3	ars-2	WBGene00000197	-0.12	0.00	-0.21	-0.41	-0.40	-0.09	-0.18	-0.20	-0.11	-0.19	0.14	9	-4.18	0.0031
tRNA-Arg	6.1.1.19	Arginyl-tRNA synthetase	C29H12.1	rrt-2	WBGene00004680	-0.23	0.30	-0.04	-0.61	-0.06	-0.19	-0.23	-0.23	-0.22	-0.17	0.24	9	-2.11	0.0683
tRNA-Arg	6.1.1.19	Arginyl-tRNA synthetase	F26F4.10	rrt-1	WBGene00004679	-0.17	-0.12	-0.24	-0.50	-0.73	-0.24	-0.36	-0.42	-0.37	-0.35	0.19	9	-5.59	0.0005
tRNA-Asp	6.1.1.12	Aspartyl-tRNA synthetase	B0464.1	drs-1	WBGene00001094	0.23	-0.02	0.02	-0.23	-0.13	0.02	-0.10	-0.13	-0.10	-0.05	0.13	9	-1.11	0.2986
tRNA-Asp	6.1.1.12	Aspartyl-tRNA synthetase	F10C2.6	drs-2	WBGene00001095	-0.20	0.11	0.01	-0.46	-0.33	-0.18	-0.28	-0.30	-0.19	-0.20	0.17	9	-3.49	0.0082
tRNA-Glu	6.1.1.17	Glutamyl-tRNA synthetase	T07A9.2	ers-3	WBGene00001338	-0.03	0.04	-0.19	-0.64	-0.14	-0.10	-0.03	-0.15	-0.07	-0.15	0.20	9	-2.20	0.0591
tRNA-Glu	6.1.1.17	Glutamyl-tRNA synthetase	ZC434.5	ers-2	WBGene00001337	-0.29	-0.11	-0.42	-0.83	-0.28	-0.04	-0.11	-0.51	-0.42	-0.33	0.25	9	-4.08	0.0035
tRNA-Glu	6.1.1.17	Glutamyl-tRNA synthetase	Y41E3.4	ers-1	WBGene00001336	-0.41	-0.16	-0.31	-0.80	-0.48	-0.20	-0.33	-0.37	-0.41	-0.39	0.19	9	-6.22	0.0003
tRNA-Gly	6.1.1.14	Glycyl-tRNA synthetase	T10F2.1	grs-1	WBGene00001744	-0.35	-0.16	-0.50	-0.66	-0.55	-0.23	-0.38	-0.45	-0.37	-0.41	0.16	9	-7.84	0.0001
tRNA-Ile	6.1.1.5	Isoleucyl-tRNA synthetase	C25A1.7	irs-2	WBGene00002153	-0.13	-0.22	-0.29	-0.12	-0.18	-0.12	-0.15	-0.10	0.03	-0.14	0.09	9	-4.85	0.0013
tRNA-Ile	6.1.1.5	Isoleucyl-tRNA synthetase	R11A8.6	irs-1	WBGene00002152	-0.24	0.24	-0.40	-0.96	-0.55	-0.04	-0.25	-0.41	-0.39	-0.33	0.33	9	-3.01	0.0168
tRNA-Leu	6.1.1.4	Leucyl-tRNA synthetase	R74.1	lrs-1	WBGene00003073	-0.04		-0.11	-0.49	-0.49	-0.09	-0.23	-0.05	-0.06	-0.20	0.19	8	-2.88	0.0237
tRNA-Leu	6.1.1.4	Leucyl-tRNA synthetase	ZK524.3	lrs-2	WBGene00003074	-0.20	0.17	-0.27	-0.90	-0.60	-0.28	-0.31	-0.43	-0.24	-0.34	0.29	9	-3.48	0.0084
tRNA-Lys	6.1.1.6	Lysyl-tRNA synthetase	T02G5.9	krs-1	WBGene00002238	-0.33	-0.20	-0.33	-0.83	-0.49	-0.13	-0.34	-0.57	-0.48	-0.41	0.21	9	-5.87	0.0004
tRNA-Phe	6.1.1.20	Phenylalanyl-tRNA synthetase	Y60A3A.13	frs-3	WBGene00013361	0.01	-0.40	-0.17	-0.40	-0.37	-0.12	-0.30	-0.13	-0.10	-0.22	0.15	9	-4.38	0.0023
tRNA-Phe	6.1.1.20	Phenylalanyl-tRNA synthetase	T08B2.9	frs-1	WBGene00001497	-0.11	-0.02	-0.28	-0.46	-0.51	-0.18	-0.40	-0.30	-0.25	-0.28	0.16	9	-5.20	0.0008
tRNA-Phe	6.1.1.20	Phenylalanyl-tRNA synthetase	F22B5.9	frs-2	WBGene00001498	-0.26	-0.06	-0.52	-0.73	-0.71	-0.18	-0.27	-0.52	-0.41	-0.41	0.23	9	-5.24	0.0008
tRNA-Tyr	6.1.1.2	Tryptophanyl-tRNA synthetase	C34E10.4	wrs-2	WBGene00006946	-0.06	0.19	-0.30	-0.99	-0.13	-0.03	-0.16	-0.38	-0.34	-0.24	0.33	9	-2.22	0.0575
tRNA-Tyr	6.1.1.1	Tyrosyl-tRNA synthetase	Y105E8A.19		WBGene00013677	no data	no data	no data	no data	-0.46	no data	no data	-0.03	-0.34	-0.28	0.22	3	-2.16	0.1634
tRNA-Tyr	6.1.1.2	Tryptophanyl-tRNA synthetase	Y80D3A.1	wrs-1	WBGene00006945	-0.53	0.57	-0.63	-1.09	-0.34	-0.02	-0.27	-0.46	-0.30	-0.34	0.45	9	-2.26	0.0534
tRNA-Val	6.1.1.9	Valyl-tRNA synthetase	ZC513.4	vrs-1	WBGene00006935	-0.16	0.38	-0.17	-0.85	-0.43	-0.14	-0.24	-0.61	-0.33	-0.28	0.34	9	-2.48	0.0383
tRNA-Val	6.1.1.9	Valyl-tRNA synthetase	Y87G2A.5	vrs-2	WBGene00006936	-0.60	0.43	-0.66	-1.58	-0.38	0.21	-0.43	-0.56	-0.59	-0.46	0.57	9	-2.44	0.0407
Phe/Tyr metabolism	1.13.11.27	4-hydroxyphenylpyruvate dioxygenase	T21C12.2	hpd-1	WBGene00001993	-0.31	-0.59	-0.81	0.44	-0.33	0.17	-0.23	-0.36	-0.55	-0.29	0.38	9	-2.23	0.0565
Phe/Tyr metabolism	1.13.11.5	homogentisate 1,2-dioxygenase	W06D4.1	hgo-1	WBGene00001843	0.49	0.26	-0.06	0.98	0.76	0.56	0.68	0.41	0.20	0.48	0.32	9	4.52	0.0020
Phe/Tyr metabolism	1.14.16.1	phenylalanine hydroxylase	K08F8.4	pah-1	WBGene00000240	0.69	0.50	0.35	0.08	1.29	0.80	1.08	0.85	0.74	0.71	0.37	9	5.81	0.0004
Phe/Tyr metabolism	1.14.17.1	dopamine beta-monooxygenase	H13N06.6	tbh-1	WBGene00006541	0.34	0.70	0.92	0.16	-0.02	-0.12	0.18	0.27	0.34	0.31	0.33	9	2.81	0.0227
Phe/Tyr metabolism	1.14.18.1	tyrosinase	C34G6.2	tyr-4	WBGene00016419	1.15	0.92	0.89	1.34	1.37	0.99	1.05	1.45	1.08	1.14	0.20	9	16.70	0.0000
Phe/Tyr metabolism	1.14.18.1	tyrosinase	C02C2.1	tyr-1	WBGene00015332	0.96	1.88	1.16	1.20	1.34	0.80	1.35	1.27	0.88	1.20	0.32	9	11.17	0.0000
Phe/Tyr metabolism	1.14.18.1	tyrosinase	K08E3.1	tyr-2	WBGene00010661	1.41	1.65	1.59	0.52	1.64	1.18	0.74	0.95	0.35	1.11	0.50	9	7.11	0.0001
Phe/Tyr metabolism	1.14.18.1	tyrosinase	F21C3.2	tyr-3	WBGene00009001	no data	no data	no data	no data	no data	no data	no data	no data	no data	no data	no data	0	no data	no data
Phe/Tyr metabolism	2.6.1.1	aspartate transaminase	C44E4.3		WBGene00016652	-0.10	-0.21	-0.29	-0.96	-0.47	-0.11	-0.46	-0.37	-0.29	-0.36	0.26	9	-4.17	0.0031
Phe/Tyr metabolism	2.6.1.1	aspartate transaminase	T01C8.4		WBGene00020145	-0.47	no data	no data	no data	no data	no data	no data	no data	no data	-0.47	-	1	-	-
Phe/Tyr metabolism	2.6.1.1	aspartate transaminase	T01C8.5		WBGene00020146	0.55	0.08	0.15	0.50	0.54	0.34	0.21	0.33	0.02	0.30	0.20	9	4.53	0.0019
Phe/Tyr metabolism	2.6.1.5	tyrosine transaminase	F42D1.2		WBGene00009628	0.66	0.44	-0.19	-1.98	0.92	0.24	0.79	0.65	0.57	0.23	0.89	9	0.78	0.4554
Phe/Tyr metabolism	3.7.1.2	fumarylacetoacetase	K10C2.4		WBGene00019620	0.55	-0.19	0.05	1.20	-0.13	0.40	0.24	0.46	0.18	0.31	0.42	9	2.19	0.0600
Phe/Tyr metabolism	4.1.1.28	DOPA decarboxylase	ZK829.2	hdl-1	WBGene00001839	-0.04	-0.15	-0.22	-0.16	0.18	0.16	-0.10	-0.07	0.03	-0.04	0.14	9	-0.88	0.4039
Phe/Tyr metabolism	4.1.1.28	DOPA decarboxylase	C05D2.3		WBGene00015467	no data	-0.08	-0.04	0.15	1.54	1.66	-0.03	0.62	0.36	0.52	0.71	8	2.09	0.0746
Phe/Tyr metabolism	5.2.1.2	maleylacetoacetate isomerase	D1053.1	gst-42	WBGene00001790	0.65	0.45	0.39	0.63	0.76	0.59	1.03	0.75	0.61	0.65	0.19	9	10.45	0.0000
Leu/Ile/Val metabolism	2.6.1.42	branched-chain amino acid aminotransferase, BCAT	Y44A6D.5		WBGene00012855	no data	no data	-0.46	no data	1.82	no data	no data	0.76	0.05	0.54	0.99	2	0.78	0.5796
Leu/Ile/Val metabolism	2.6.1.42	branched-chain amino acid aminotransferase,BCAT	K02A4.1	bcat-1	WBGene00001149	0.58	-0.19	-0.04	-0.34	0.33	0.37	0.29	-0.03	0.09	0.12	0.30	9	1.19	0.2677
Leu/Ile/Val metabolism	1.2.4.4	BCKD, E1, decarboyxlase, alpha subunit	Y39E4A.3		WBGene00012713	-0.16	-0.59	-0.30	0.14	-0.29	-0.07	-0.34	-0.22	-0.31	-0.24	0.20	9	-3.55	0.0075
Leu/Ile/Val metabolism	1.2.4.4	BCKD, E1, decarboyxlase, beta subunit	F27D4.5	tag-173	WBGene00006518	-0.10	-0.33	-0.34	0.16	-0.45	-0.01	-0.30	-0.49	-0.45	-0.26	0.22	9	-3.43	0.0089
Leu/Ile/Val metabolism	23.1.168	BCKD, E2, dihydrolipoamide acyltransferase	ZK669.4		WBGene00014054	-0.27	-0.29	-0.41	-0.61	-0.25	-0.10	-0.44	-0.38	-0.47	-0.36	0.15	9	-7.23	0.0001
Leu/Ile/Val metabolism	1.8.1.4	BCKD, E3, dihydrolipoamide dehydrogenase	LLC1.3		WBGene00010794	-0.10	-0.26	-0.16	-0.08	-0.04	0.13	-0.28	-0.20	-0.21	-0.13	0.13	9	-3.13	0.0140
Leu/Ile/Val metabolism	1.3.99.3	acyl-CoA dehydrogenase	K05F1.3	acdh-8	WBGene00019406	0.12	-1.16	-1.31	-0.15	1.00	2.13	-0.43	-0.05	-0.38	-0.03	1.06	9	-0.07	0.9440
Leu/Ile/Val metabolism	1.3.99.3	acyl-CoA dehydrogenase	K09H11.1		WBGene00019599	0.10	-0.32	-0.31	0.26	0.53	0.17	0.24	0.16	-0.19	0.07	0.29	9	0.74	0.4788
Leu/Ile/Val metabolism	1.3.99.3	acyl CoA dehydrogenase	T25G12.5	acdh-7	WBGene00020812	no data	no data	no data	no data	no data	no data	no data	no data	no data	-	-	-	-	-
Leu/Ile/Val metabolism	1.399.10	isovaleryl-CoA dehydrogenase	C02B10.1	ivd-1	WBGene00006518	-0.10	-0.33	-0.34	0.16	-0.45	-0.01	-0.30	-0.49	-0.45	-0.26	0.22	9	-3.43	0.0089
Leu/Ile/Val metabolism	1.399.10	isovaleryl-CoA dehydrogenase	C02D5.1	acdh-6	WBGene00015335	0.17	0.29	0.96	-1.36	0.76	-0.26	0.36	0.53	0.59	0.23	0.69	9	0.98	0.3542
Leu/Ile/Val metabolism	4.2.1.17	enoyl-CoA hydratase	C29F3.1	ech-1	WBGene00001150	-0.18	0.44	-0.12	-0.38	0.06	-0.03	-0.01	-0.13	-0.13	-0.05	0.22	9	-0.72	0.4934
Leu/Ile/Val metabolism	4.2.1.17	enoyl-CoA hydratase	T05G5.6	ech-6	WBGene00001155	-0.22	-0.60	-0.47	0.62	-0.28	0.04	-0.33	-0.04	-0.21	-0.17	0.35	9	-1.40	0.1980
Leu/Ile/Val metabolism	4.2.1.17	enoyl-CoA hydratase	F56B3.5	ech-5	WBGene00001154	-0.34	0.21	-0.82	-0.39	-0.56	-0.09	-0.50	-0.45	-0.20	-0.35	0.30	9	-3.53	0.0077
Leu/Ile/Val metabolism	4.2.1.17	enoyl-CoA hydratase	F38H4.8	ech-2	WBGene00001151	-0.33	-0.04	-0.54	-0.64	-0.23	-0.03	-0.23	-0.54	-0.43	-0.33	0.22	9	-4.55	0.0019
Leu/Ile/Val metabolism	4.2.1.17	enoyl-CoA hydratase	F43H9.1	ech-3	WBGene00001152	0.35	0.12	0.26	0.77	0.09	-0.03	0.26	0.31	0.23	0.26	0.22	9	3.50	0.0081
Leu/Ile/Val metabolism	4.2.1.17	enoyl-CoA hydratase	Y105E8A.4	ech-7	WBGene00001156	0.50	0.38	0.04	0.89	1.14	0.45	0.72	0.80	0.35	0.59	0.33	9	5.28	0.0007
