# Supplementary material for: A metabolic signature of long life in Caenorhabditis elegans
Source: BMC Biol. 2010 Feb 10;8:14. doi: 10.1186/1741-7007-8-14 (PMC2829508; doi:10.1186/1741-7007-8-14)
Supplement: Additional file 3 — Table S3. List of assigned metabolites detected in worm samples by 1D NMR, and associated resonance chemical shifts and multiplicity. Only the most characteristic resonances are listed in the table. [file 1741-7007-8-14-S3.PDF]

SI Table 3

Compound class	Compound name	NMR resonances	Fitted
Amino acid	Alanine	1.48d	x
Amino acid	Arginine	3.25t	x
Amino acid	Asparagine	2.96ABX, 2.87ABX	
Amino acid	Aspartate	2.82ABX, 2.68ABX	x
Amino acid	Cystathionine	2.73m	
Amino acid	Glutamine	2.46q, 2.14m	
Amino acid	Glutamate	2.36m, 2.14m, 2.07m	x
Amino acid	Glycine	3.56s	x
Amino acid	Histidine	7.15s*	
Amino acid	Hydroxyproline†	3.48dd, 3.36, 2.44m	x
Amino acid	Isoleucine	1.02d, 0.95t	x
Amino acid	Leucine	1.70m, 0.97d, 0.96d	x
Amino acid	Lysine	3.03t, 1.92m, 1.73m	x
Amino acid	Methionine	2.65t, 2.14s	
Amino acid	Phenylalanine	7.43t, 7.38t, 7.33d	x
Amino acid	Phosphoserine†	4.19m, 4.07dt, 3.95dd	x
Amino acid	Serine	3.99ABX, 3.95ABX	
Amino acid	Threonine	4.26m, 3.59d, 1.33d	
Amino acid	Tryptophan	7.74d, 7.55d, 7.33s, 7.29t, 7.21t	x
Amino acid	Tyrosine	7.20d, 6.91d	x
Amino acid	Valine	1.05d, 1.00d	x
Organic acid	Acetate	1.92s	x
Organic acid	Formate	8.46s	
Organic acid	Fumarate	6.52s	
Organic acid	Lactate	4.11q, 1.33d	x
Organic acid	Malate	4.30dd, 2.67ABX, 2.36ABX	x
Organic acid	Nicotinate	8.94sb, 8.62dd, 8.26dt, 7.53dd	x
Organic acid	Propanoate	2.18q, 1.06t	x
Organic acid	Succinate	2.41s	x
Nucleoside	Adenosine	6.08d	
Nucleoside	Inosine	6.11d	
Nucleotide	NAD	9.34s, 9.15d, 8.84d, 8.43s, 8.18s, 6.09d, 6.04d	x
Carbohydrate	Glucose	5.24d, 4.65d	x
Carbohydrate	Trehalose	5.20d, 3.65dd, 3.46t	x
Phospholipid-related	Betaine	3.91s, 3.27s	x
Phospholipid-related	Choline	4.05m, 3.52m, 3.21s	x
Phospholipid-related	GPC	4.33m, 3.62ABX, 3.24s	x
Phospholipid-related	Glycerol	3.65ABX, 3.56ABX	x
Phospholipid-related	Phosphocholine	4.18m, 3.60m, 3.23s	x
Polyamine	Putrescine	3.06m, 1.78m	

*Resonance is pH sensitive and shifts between

†Only observed in dauers
